# Two nucleotide sugar transporters are important for cell wall integrity and full virulence of *Magnaporthe oryzae*

**DOI:** 10.1111/mpp.13304

**Published:** 2023-02-12

**Authors:** Deng Chen, Muhammad Kamran, Shen Chen, Junjie Xing, Zhiguang Qu, Caiyun Liu, Zhiyong Ren, Xuan Cai, Xiao‐Lin Chen, Jingbo Xu

**Affiliations:** ^1^ State Key Laboratory of Hybrid Rice Hunan Hybrid Rice Research Center Changsha China; ^2^ State Key Laboratory of Agricultural Microbiology, Provincial Key Laboratory of Plant Pathology of Hubei Province College of Plant Science and Technology, Huazhong Agricultural University Wuhan China; ^3^ Guangdong Provincial Key Laboratory of High Technology for Plant Protection Plant Protection Research Institute, Guangdong Academy of Agricultural Sciences Guangzhou China

**Keywords:** appressorium, cell wall saccharides, *Magnaporthe oryzae*, sugar transporter, sugar utilization

## Abstract

Cell wall polysaccharides play key roles in fungal development, virulence, and resistance to the plant immune system, and are synthesized from many nucleotide sugars in the endoplasmic reticulum (ER)‐Golgi secretory system. Nucleotide sugar transporters (NSTs) are responsible for transporting cytosolic‐derived nucleotide sugars to the ER lumen for processing, but their roles in plant‐pathogenic fungi remain to be revealed. Here, we identified two important NSTs, *NST1* and *NST2*, in the rice blast fungus *Magnaporthe oryzae*. Both NSTs were localized in the ER, which was consistent with a function in transporting nucleotide sugar for processing in the ER. Sugar transport property analysis suggested that NST1 is involved in transportation of mannose and glucose, while NST2 is only responsible for mannose transportation. Accordingly, deletion of *NST*s resulted in a significant decrease in corresponding soluble saccharides abundance and defect in sugar utilization. Moreover, both *NST*s played important roles in cell wall integrity, were involved in asexual development, and were required for full virulence. The *NST* mutants exhibited decreasing external glycoproteins and exposure of inner chitin, which resulted in activation of the host defence response. Altogether, our results revealed that two sugar transporters are required for fungal cell wall polysaccharides accumulation and full virulence of *M. oryzae*.

## Graphical abstract

Nucleotide sugar transporters NST1 and NST2 are involved in the transportation of different sugars and are required for cell wall polysaccharide accumulation and immune evasion of *Magnaporthe oryzae*.

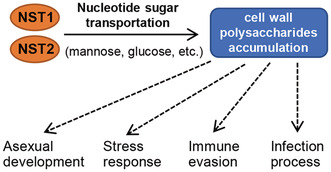

## 1 INTRODUCTION

Rice blast is a devastating disease that threatens the production and quality of rice worldwide (Dean et al., 2012). *Magnaporthe oryzae*, the causal agent of rice blast, has been used as a model microorganism to study the molecular mechanism of interaction between host plants and pathogens (Ebbole, 2007). The cell wall, as the most external structure, provides sufficient mechanical support for development, infection, and adaptation to environmental stresses. It also plays crucial roles in interaction with host cells (Arana et al., 2009; Bowman & Free, 2006). Structural carbohydrate components of the fungal cell wall include chitin, glucan, and glycoproteins (Latgé, 2007, 2010), which are mainly biosynthesized by glycosyltransferases using nucleotide sugars as substrates (Arana et al., 2009; Scheible & Pauly, 2004). This biological process usually take place in the endoplasmic reticulum (ER)–Golgi secretory pathway while nucleotide sugars are synthesized primarily in the cytosol. To overcome this spatial partitioning between enzymes and substrates, nucleotide sugar transporters (NSTs) exist to transport nucleotide sugars from the cytosol into the ER lumen (Doering, 2009; Ebert et al., 2015; Li et al., 2018a).

NSTs are members of the NST‐triose phosphate translocator (TPT) superfamily widely present in eukaryotes (Ebert et al., 2015; Knappe et al., 2003). NSTs have their own protein features, possessing 300 to 400 amino acids with six to 10 transmembrane domains. NSTs contain a large group of proteins owing to the collocations of various nucleotides (e.g., UMP [uridine monophosphate], UDP [uridine diphosphate], CMP [cytidine monophosphate], GDP [guanosine diphophate], ADP [adenosine diphosphate], AMP [adenosine monophosphate]) and different kinds of sugars (e.g., glucose, galactose, mannose, rhamnose, xylose, fucose). Phylogenetic analyses revealed more than 50 NSTs in *Arabidopsis thaliana* that are classified into six clades (Ebert et al., 2015; Rautengarten et al., 2014). Given the difficulty of predicting the substrate specificity of NSTs and adequate availability of nucleotide sugar substrates, only a few plant NSTs have been functionally characterized, specifically those responsible for the transport of GDP‐mannose, UDP‐galactose, and UDP‐glucose (Rautengarten et al., 2014; Zhao et al., 2018). While blocking of the biosynthesis of nucleotide sugar substrates such as GDP‐fucose leads to developmental and biochemical defects (O'Neill et al., 2001), mutation of GDP‐mannose transporters only resulted in observable phenotypes in *Arabidopsis* (Mortimer et al., 2013). With a novel biochemical approach developed to analyse NST activities and their substrate specificity (Ito et al., 2014), a family of UDP‐rhamnose/UDP‐galactose transporters has been identified in *Arabidopsis* and one of them was found to be important for mucilage formation during seed development (Rautengarten et al., 2014). A UDP‐xylose transporter (UXT) family containing three UXT proteins was characterized to have the capacity to transport UDP‐xylose as well as a small amount of UDP‐arabinopyranose. Cell wall composition analyses indicate apparently decreased content in xylose‐related polysaccharides including xylan and glucuronoxylan but enriched content in proteoglycans in *UXT* deletion mutants compared to the wild type (Ebert et al., 2015). A human NST SLC25B4 has been identified to be a UDP‐xylose transporter that controls both UDP‐glucuronic acid and UDP‐xylose production (Bakker et al., 2009). SLC35B4 is another human NST that encodes a Golgi‐localized NST with dual specificity for UDP‐xylose and UDP‐N‐acetylglucosamine (Ashikov et al., 2005).

In fungi, NSTs have been investigated in *Cryptococcus neoformans*, the leading cause of fungal meningitis and responsible for hundreds of thousands of deaths worldwide (Doering, 2009; Li et al., 2020a). Two UDP‐xylose transporters have been characterized in *C. neoformans* with different subcellular localizations, expression patterns and kinetic parameters, but both are required for xylose incorporation to the polysaccharide capsule and are critical for fungal virulence (Li et al., 2018a). Another study demonstrated that xylose transport is modulated by *C. neoformans* to evade pulmonary immunity (Li et al., 2020a). Moreover, UDP‐glucuronic acid transport was also proved to be essential for capsule synthesis, environmental stress response, and virulence in *C. neoformans* (Li et al., 2018b). The UDP‐galactose transporter UGT1 was found to exhibit transportation activity of both UDP‐galactose and UDP‐acetylgalactosamine. Deletion of *UGT1* results in growth defects, altered capsules, and abnormal cellular morphology, as well as lack of virulence to mouse (Li et al., 2017).

Sugar transporters have been identified in plant‐pathogenic fungi including *Uromyces fabae*, *Ustilago maydis*, *Puccinia striiformis*, *Colletotrichum graminicola*, and *Colletotrichum higginsianum* (Chang et al., 2020; Schuler et al., 2015; Voegele et al., 2001; Wahl et al., 2010; Yuan et al., 2021). The sucrose transporter Srt1 exhibits unusually high sucrose affinity and allows direct utilization of sucrose without extracellular hydrolysis into monosaccharides. Efficient competition of the *U. maydis* Srt1 protein with the low‐affinity plant sucrose transporters for apoplastic sucrose can protect pathogens from pathogen‐associated molecular patterns (PAMP)‐triggered immunity (PTI) (Wahl et al., 2010). Another monosaccharide transporter, a hexose transporter Hxt1, was characterized as a high‐affinity transporter for glucose, fructose, and mannose. Deletion of *U. maydis HXT1* results in significantly reduced growth and decreased virulence to maize (Schuler et al., 2015). In the rust fungus *Ustilago fabae*, *HXT1* possess transport activities for glucose and fructose, enabling the pathogen to compete for host nutrients (Voegele et al., 2001). In another rust fungus, *P. striiformis*, *HXT1* is responsible for sugar uptake as well as the pathogenicity of wheat stripe rust (Chang et al., 2020). Similarly, *ChHXT6* encodes a hexose transporter required for hexose uptake and the virulence of *C. higginsianum* (Yuan et al., 2021). These studies were mainly focused on pure sugar transporters. However, the role of nucleotide sugar transporters in carbohydrate metabolism and the virulence of plant‐pathogenic fungi remains largely unknown.

In this study, two NST proteins homologous to the UDP‐xylose transporters of *C. neoformans* were characterized in *M. oryzae*. We found that NST1 transported nucleotide mannose and glucose, while NST2 only transported nucleotide mannose. Deletion of single or double *NST*s led to a significant decrease in the abundance of specific saccharides and utilization of certain sugars. Therefore, the *NST* deletion mutants were defective in cell wall integrity and this consequently affected the stress response, growth, conidiation, and appressorial adhesion, as well as immune evasion due to exposure of cell wall chitin. Taken together, our results reveal the important roles of two nucleotide sugar transporters in the development and infection of *M. oryzae*.

## 2 RESULTS

### 2.1 Identification of two NSTs in *M. oryzae*

**FIGURE 1:**
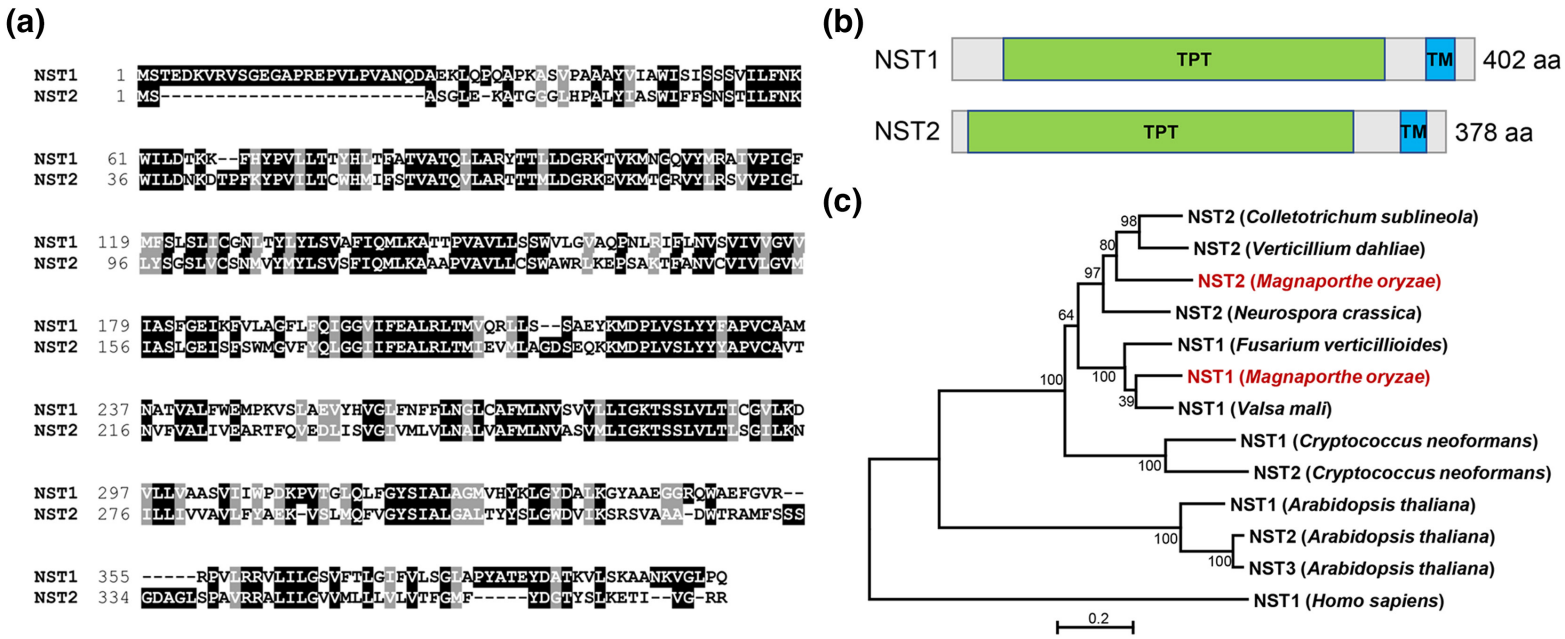
NST1 and NST2 show similarities in *Magnaporthe oryzae* and are conserved in different organisms. (a) Amino acid sequence alignment between NST1 and NST2 in *M. oryzae*. (b) Protein domains of *M. oryzae* NST1 and NST2. aa, amino acid; TPT, triose‐phosphate transporter family domain; TM, transmembrane domain. (c) Phylogenetic analysis of NSTs between different species.

Using two UDP‐xylose transporter proteins of *C. neoformans* to search possible homologues in the *M. oryzae* genome (http://fungi.ensembl.org/Magnaporthe_oryzae/Info/Index) (Li et al., 2018a), we found a pair of proteins, MGG_06694 and MGG_04030, with the highest homologies with two query UDP‐xylose transporters. We designated *MGG_06694* and *MGG_04030* as *NST1* and *NST2* (*N*ucleotide *S*ugar *T*ransporter 1 and 2), respectively. *NST1* and *NST2* show 55% identities and 72% positives in amino acid sequence (Figure 1a). The NST1 and NST2 proteins were predicted to be composed of 402 and 378 amino acid residues that contain both a triose‐phosphate transporter (TPT) family domain and a transmembrane domain (Figure 1b). Phylogenetic analysis indicated that homologues of NST1 and NST2 are widely distributed and well‐conserved among eukaryotes, including in humans (*Homo sapiens*), plants (*Arabidopsis thaliana*), and fungi (*C. neoformans*, *Fusarium verticillioides*, *Neurospora crassica*, *Verticillium dahliae*) (Figure 1c).

**FIGURE 2:**
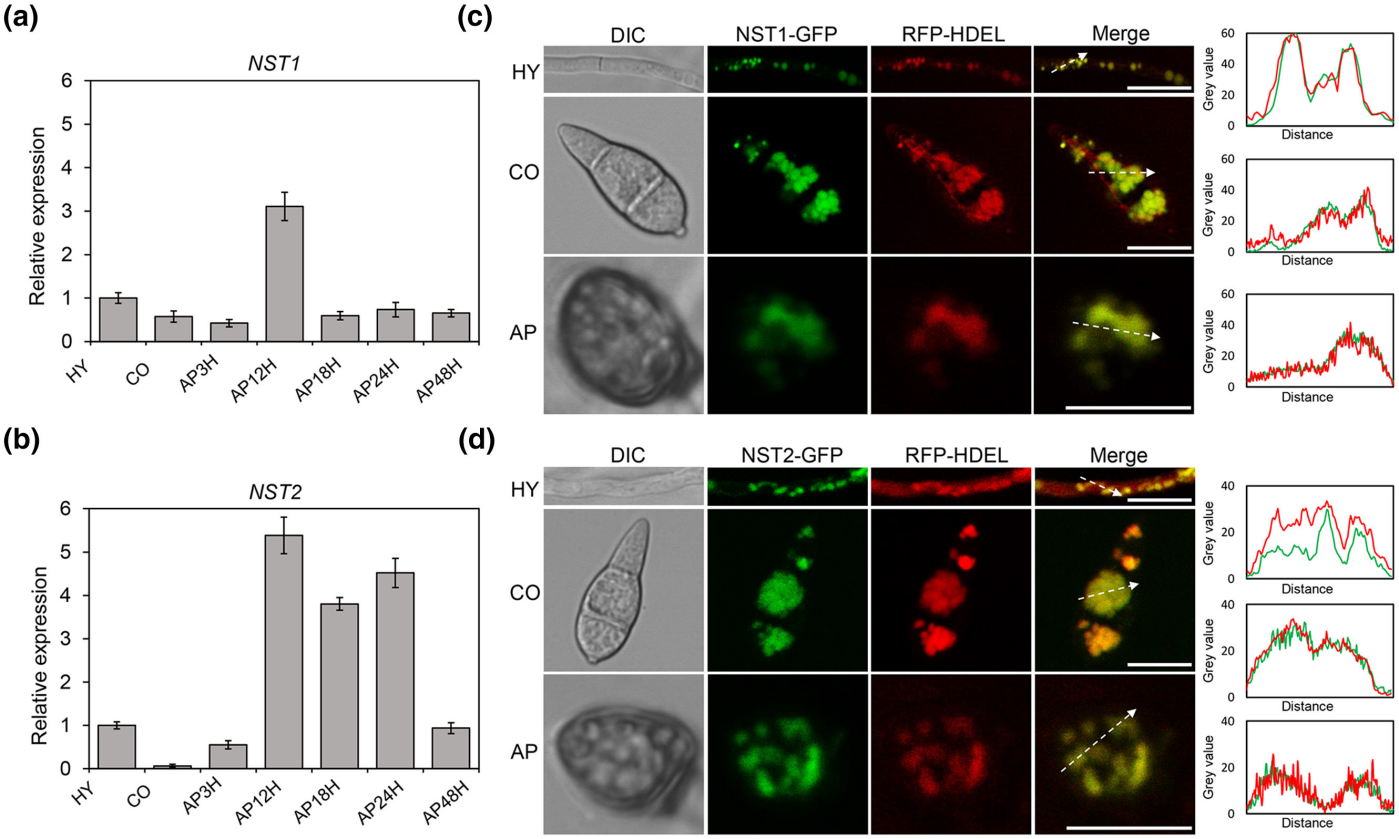
Gene expression pattern and subcellular localization of NST1 and NST2 in *Magnaporthe oryzae*. (a) Relative expression of *NST1* at different developmental stages. (b) Relative expression of *NST2* at different developmental stages. For (a) and (b), conidia produced from oatmeal agar (OTA) plates, appressoria at 3 and 12 h postinoculation (hpi) on Teflon films, and invasive hyphae at 18, 24, and 48 hpi in barley epidermis were harvested to extract total RNA for preparing cDNA templates and performing real‐time quantitative PCR analysis. (c) Subcellular localization of NST1. A transformant that expressed both NST1‐GFP and endoplasmic reticulum (ER)‐localized RFP‐HDEL was observed under a confocal microscope. Line‐scan graph analysis showed that NST1‐GFP was colocalized with RFP‐HDEL. Bar, 10 μm. (d) Subcellular localization of NST2. A transformant that expressed both NST2‐GFP and ER‐localized RFP‐HDEL was observed under a confocal microscope. Line‐scan graph analysis showed that NST2‐GFP was colocalized with RFP‐HDEL. Bar, 10 μm. HY, hypha; CO, conidium; AP, appressoriu; AP3H, appressoria at 3 hpi; AP12H, appressoria at 12 hpi; IH18H, infectious hyphae at 18 hpi; IH24H, infectious hyphae at 24 hpi; IH48H, infectious hyphae at 48 hpi.

To understand the possible biological function of the two NSTs in *M. oryzae*, we investigated the gene expression profiles of *NST1* and *NST2*. Samples of various developmental/infection stages including hyphae (HY), conidia (CO), immature appressoria at 3 h postinoculation (hpi, AP3H), mature appressoria at 12 hpi (AP12H), and infection hyphae at 18 hpi (IH18H) and 24 hpi (IH24H) during early invasive growth, and at 48 hpi (IH48H) during late invasive growth were collected to extract total RNA and then reverse transcribed into cDNA. Real‐time quantitative PCR (qPCR) was performed to measure expression of *NST1* and *NST2* during the above stages. We found that *NST1* was highly expressed in mature appressoria (AP12H), more than three times as much as in other stages. *NST2* was highly expressed in mature appressoria (AP12H) and the early invasive hyphal stage (IH18H and IH24H) (Figure 2a,b). These data suggest that *NST1* and *NST2* may play a role in appressoria or early invasive hyphal stages of *M. oryzae*.

### 2.2 NST1 and NST2 are both localized in the ER

NSTs were considered to transport nucleotide sugars from the cytosol into the ER lumen. We used an ER‐located marker protein, which expresses an ER‐retention signal peptide HDEL (His‐Asp‐Glu‐Leu) at the C‐terminus of red fluorescent protein (RFP), to verify the ER localization of NST1 and NST2 in *M. oryzae*. When NST1‐GFP and the ER‐localized marker protein RFP‐HDEL were cotransformed into the wild‐type P131, clear ER‐like punctae were distributed as both green and red signals in vegetative hyphae, conidia, and appressoria (Figure 2c). Similarly, when NST2‐GFP and RFP‐HDEL were cotransformed into the wild type, an obvious colocalization signal in the ER was also observed at all tested stages (Figure 2d). This result demonstrates that NST1 and NST2 are both ER‐localized proteins, which is consistent with their putative function in nucleotide sugar transportation from the cytosol into the ER lumen.

### 2.3 Deletion of *NST1* and *NST2* in *M. oryzae*

To further determine the biological function of *NST1* and *NST2*, we knocked out the two genes in the wild‐type strain P131 using a split‐PCR strategy (Figure S1a). Two *NST1* deletion mutants and two *NST2* deletion mutants were obtained after the PCR‐mediated identification (Figure S1b,c and Table S1). One of the *NST1* deletion mutants, NST1KO1, was designated as Δ*nst1* and used in the following experiments. Likewise, one of the two *NST2* deletion strains, NST2KO1, was designated as Δ*nst2* and chosen for further study. To obtain the double‐deletion mutants of *NST1* and *NST2*, *NST2* was deleted based on the Δ*nst1* single‐deletion mutant using a similar method (Figure S1d and Table S1). Two double‐deletion mutants were generated after the PCR‐mediated identification (Figure S1e). One of them, Δ*nst1*Δ*nst2*, was selected for further study. Complementary strains of Δ*nst1* and Δ*nst2* were also obtained by transforming the native promoter‐driven coding region of each gene into the corresponding single‐deletion mutant (Table S1). All tested biological phenotypes of the complementation strains were recovered into the wild type, as described below.

### 2.4 Contents of soluble sugars were changed in the *nst* mutants

**FIGURE 3:**
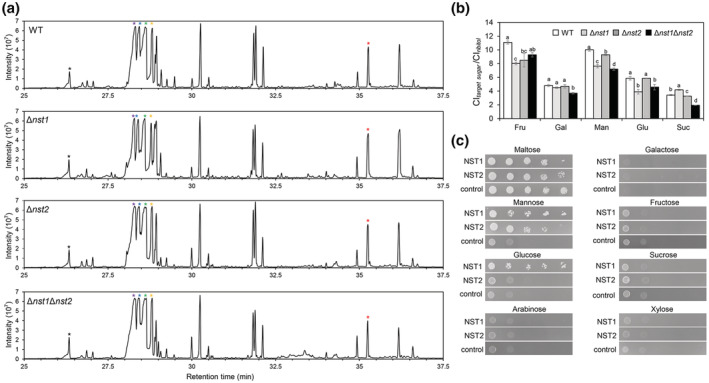
NSTs affect relative content of several cell wall saccharides. (a) Chromatograms of the wild type (WT), Δ*nst1*, Δ*nst2*, and Δ*nst1*Δ*nst2* tested by gas chromatography–mass spectrometry. Black, purple, blue, green, yellow, and red asterisks indicate the peaks of ribitol, fructose, galactose, mannose, glucose, and sucrose, respectively. (b) Statistical analysis of the chromatographic integral (CI) of a target sugar normalized to the CI of ribitol (CI *target sugar*/CI *ribitol*) among the indicated strains (one‐way analysis of variance, *p* < 0.05). Fru, fructose; Gal, galactose; Man, mannose; Glu, glucose; Suc, sucrose. (c) NST1 and NST2 were involved in the transportation of different sugars. Gradient concentration (10^7^, 10^6^, 10^5^, 10^4^, and 10^3^ cells/ml) of the yeast strain that expressed NST1, NST2, or the empty vector pDR195 (control) were dripped onto YNB medium containing a different single sugar (maltose, mannose, glucose, arabinose, galactose, fructose, sucrose, or xylose) for 2 days' incubation.

Because cell wall polysaccharides are composed of distinct sugars transported by NSTs, we analysed several monosaccharides and disaccharides among wild type (WT), Δ*nst1*, Δ*nst2*, and Δ*nst1*Δ*nst2*. Soluble sugars were extracted from isolated cell walls of these strains and analysed by gas chromatography–mass spectrometry (GC–MS) analysis. With reference to the retention time of the target peak of distinct sugar standards and the internal standard ribitol (Figure S2), we tested fructose, galactose, mannose, glucose, and sucrose in the four samples. The relative chromatographic integral (CI *target sugar*/CI *ribitol*), which represents the relative content of the target sugars, of fructose and mannose in the WT was significantly larger than for the *nst* deletion mutants. The CI ratios of galactose, mannose, glucose, and sucrose of Δ*nst1*Δ*nst2* were lowest, indicating a relatively lower sugar content in the mutants. Moreover, the CI ratios of mannose and glucose in Δ*nst2* were larger than those in Δ*nst1*, showing that *NST1* might be more responsible for transporting mannose and glucose (Figure 3a,b). This result demonstrates that NSTs affect the content of cell wall soluble sugars in *M. oryzae*.

### 2.5 NST1 and NST2 were involved in the transportation of different sugars

To determine what kinds of sugars can be transported by NST1 or NST2, the coding regions of both genes were separately inserted into yeast expression vector pDR195. The subsequent vectors were expressed in hexose transport‐deficient yeast strain EBY.VW4000. Heterologous expression in EBY.VW4000, which lacks all identified 20 hexose transporters (Chang et al., 2020), was conducted to verify whether NST1 or NST2 can complement the function of the corresponding hexose transporter. The transformed strains were diluted to different concentrations and spotted onto yeast nitrogen base (YNB) medium containing different sugars, including maltose, manose, glucose, arabinose, fructose, galactose, sucrose, or xylose, for 2 days' incubation. The strain transformed with the empty vector could only grow on the YNB medium added with maltose. The yeast strain expressing NST1 could grow on the medium in which mannose or glucose was the single carbon source, indicating that NST1 could transport these two sugars. The yeast strain expressing NST2 could grow on the medium with the addition of mannose rather than other sugars, suggesting that NST2 exhibited a substrate preference for mannose (Figure 3c). Thus, NST1 is capable of transporting glucose and mannose, while NST2 is only able to transport mannose.

### 2.6 NSTs are involved in cell wall integrity

**FIGURE 4:**
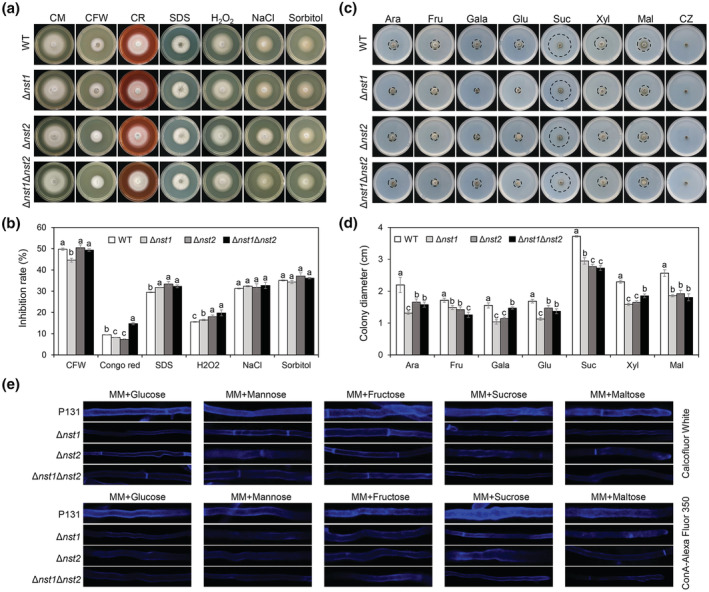
NSTs are responsible for cell wall integrity and sugar utilization. (a) Colonies of P131, Δ*nst1*, Δ*nst2*, and Δ*nst1*Δ*nst2* grown on complete medium (CM) supplemented with 0.1 mg/ml Calcofluor white (CFW), 0.2 mg/ml Congo red (CR), 0.005% sodium dodecyl sulphate (SDS), 10 mM H_2_O_2_, 0.7 M NaCl, or 1 M sorbitol for 5 days. (b) Statistical analysis of the inhibition rate of colony growth in (a) (one‐way analysis of variance [ANOVA], *p* < 0.05). (c) Colonies of wild type (WT), Δ*nst1*, Δ*nst2*, and Δ*nst1*Δ*nst2* grown on Czapek's agar (CZ) supplemented with 2% (wt/vol) arabinose (Ara), fructose (Fru), galactose (Gala), glucose (Glu), sucrose (Suc), xylose (Xyl), or maltose (Mal). (d) Statistical analysis of colony size in (d) (one‐way ANOVA; *p* < 0.05). (e) Cell wall integrity detection by CFW and ConA‐Alexa Fluor 350 staining. The strains of P131, Δ*nst1*, Δ*nst2*, and Δ*nst1*Δ*nst2* grown in minimal medium supplemented with different sugars were used for staining. The stained strains were observed using a confocal microscope.

Because *NST1* and *NST2* encoded sugar transporters that are putatively required for cell wall polysaccharide biogenesis, we tested whether the cell wall integrity of the *nst* mutants was affected. Cell wall integrity perturbing agents (Calcofluor white [CFW], Congo red [CR], and sodium dodecyl sulphate [SDS]) and osmotic pressure agents (NaCl and sorbitol) were separately added to complete medium (CM) plates for testing. When treated with CR, the inhibition ratio of Δ*nst1*Δ*nst2* was significantly higher than that of the WT, even though no difference was detected between the single‐deletion mutant and the WT. Compared with the WT, both the single‐ and double‐deletion mutants were more sensitive to SDS. When treated with CFW, Δ*nst1* exhibited a lower inhibition ratio, but no significant difference was found among WT, Δ*nst2*, and Δ*nst1*Δ*nst2*. No difference was detected between the mutants and WT when added to plates with NaCl or sorbitol (Figure 4a,b). Therefore, *NST1* and *NST2* are important for cell wall integrity but dispensable for osmotic pressure response.

### 2.7 Deleting *NST*s leads to growth defects in different sugar conditions

Because *NST1* and *NST2* are required for sugar transportation, we assumed that under different sugar supplying conditions, the cell wall polysaccharides biogenesis could be affected in *nst* mutants, leading to defective fungal growth. To test this possibility, Czapek's agar (CZ), which provides base inorganic salts, was supplemented with various sugars to test fungal growth. After 5 days, colony growth of the *nst* single mutant or double mutant was slower than WT in CZ medium with arabinose, fructose, galactose, glucose, sucrose, xylose, or maltose. The strains barely grew in the CZ medium without any sugar addition (Figure 4c,d). To determine whether the cell wall integrity of the *nst* mutants was affected under different sugar supply conditions, we stained the cell wall with CFW and Alexa Fluor 350‐fused lectin concanavalin A (ConA‐Alexa Fluor 350). CFW binds nonspecifically to β‐linked‐polysaccharide polymers, such as β‐glucans, which are one of the main components in fungal cell wall. ConA recognizes polysaccharide structures whose terminal residues are α‐d‐mannose and α‐d‐glucose (Brodsky et al., 2000), which can be used to detect highly mannosylated cell wall structures. The results showed that in minimal medium with glucose, mannose, fructose, or sucrose as the sole sugar, weaker fluorescence of CFW and ConA‐Alexa Fluor 350 in the cell wall region of the *nst* mutants was observed (Figure 4e), suggesting that the cell wall structures of the mutants were severely defective.

### 2.8 NST1 and NST2 are important for asexual development

**FIGURE 5:**
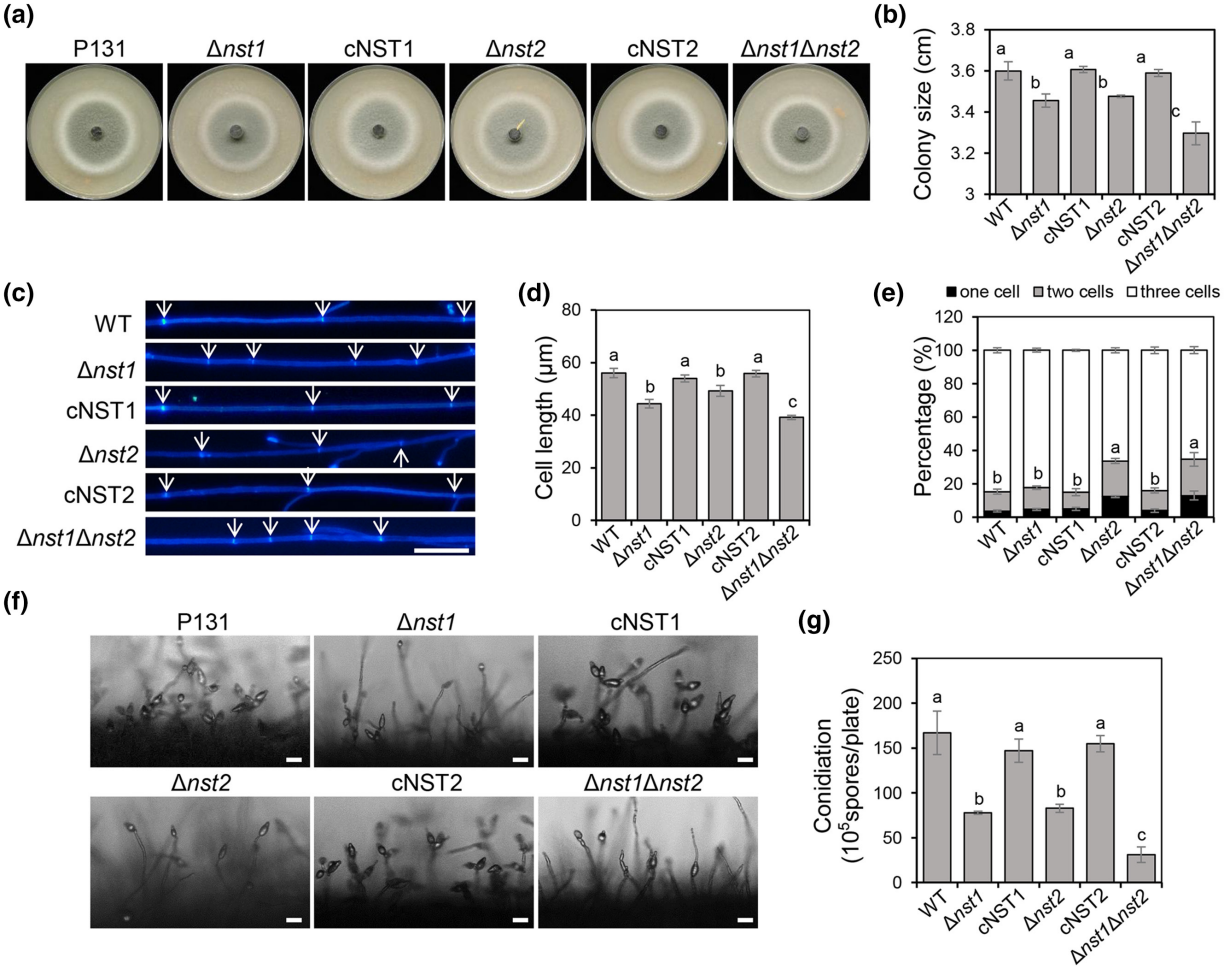
NSTs are important for asexual development of *Magnaporthe oryzae*. (a) Colonies of wild type (WT), Δ*nst1*, cNST1, Δ*nst2*, cNST2, and Δ*nst1*Δ*nst2* grown on oatmeal agar plates for 5 days. (b) Statistical analysis of the colony diameters of the indicated strains (one‐way analysis of variance [ANOVA], *p* < 0.05). (c) Hyphal tips of WT, Δ*nst1*, cNST1, Δ*nst2*, cNST2, and Δ*nst1*Δ*nst2* were stained by Calcofluor white (CFW). White arrows show septa of hyphae. Bar, 20 μm. (d) Statistical analysis of the near‐apical hyphal tip cell length of the indicated strains (one‐way ANOVA, *p* < 0.05). (e) Statistical analysis of percentages of conidia with three cells, two cells, and one cell (one‐way ANOVA, *p* < 0.05). (f) Conidiophores of WT, Δ*nst1*, cNST1, Δ*nst2*, cNST2, and Δ*nst1*Δ*nst2* were observed under a microscope. Bar, 20 μm. (g) Statistical analysis of conidiation of the indicated strains (one‐way ANOVA, *p* < 0.05). WT, wild‐type strain P131; cNST1, *NST1* complementary strain Δ*nst1/NST1*; cNST2, *NST2* complementary strain Δ*nst2/NST2*; Δ*nst1*, *NST1* deletion mutant; Δ*nst2*, *NST2* deletion mutant; Δ*nst1*Δ*nst2*, *NST1* and *NST2* double‐deletion mutant.

As cell wall integrity is important for fungal growth and development, we tested the colony growth and conidia production of the *nst* mutants. When cultured on oatmeal agar (OTA) plates for 5 days, the colony diameter of WT was 3.6 cm. In comparison, the diameters of Δ*nst1* and Δ*nst2* were both less than 3.5 cm, slightly smaller than the WT or the complemented strains cNST1 and cNST2. The size of Δ*nst1*Δ*nst2* was around 3.3 cm, significantly smaller than any single‐deletion mutant (Figure 5a,b). We then observed the hyphal tip growth of these strains. CFW was applied to stain the septa of subapical hyphal cells. The average subapical hyphal cell length of WT, cNST1, and cNST2 was evidently longer than that of Δ*nst1* or Δ*nst2*. Strikingly, the average length of the Δ*nst1*Δ*nst2* hyphal cell was even less than that of Δ*nst1* and Δ*nst2* (Figure 5c,d). This result is consistent with the colony growth on the OTA plate. Therefore, both *NST1* and *NST2* are important for vegetative growth of *M. oryzae*.

CFW was also used to stain septa of conidia to observe conidial morphology among these strains. More than 80% of WT, Δ*nst1*, cNST1, and cNST2 conidia had two septa, whereas around 70% of Δ*nst2* or Δ*nst1*Δ*nst2* conidia exhibited three‐cells morphology. The percentage of two‐cells conidia of WT or Δ*nst1* was about 10%; in contrast, nearly 20% of Δ*nst2* or Δ*nst1*Δ*nst2* conidia had two cells (Figure 5e). These data indicate that *NST2*, but not *NST1*, is important for conidial morphology.

Furthermore, conidiophore was observed and conidiation was measured to evaluate if NSTs play a role in conidia production. Clusters of conidia were observed on almost every conidiophore of WT, cNST1, and cNST2 strains; in comparison, sparse conidia were produced on the conidiophores of Δ*nst1* and Δ*nst2*, and only one or two conidia were observed on the Δ*nst1*Δ*nst2* conidiophore (Figure 5f). The WT strain produced almost twice as many conidia as produced by Δ*nst1* or Δ*nst2*. The conidiation of Δ*nst1*Δ*nst2* was even less than that of the single‐deletion mutants (Figure 5g). Thus, *NST1* and *NST2* are both important for asexual development, including vegetative growth, conidial morphology, and conidiation.

### 2.9 NST1 and NST2 are important for full virulence of *M. oryzae*

**FIGURE 6:**
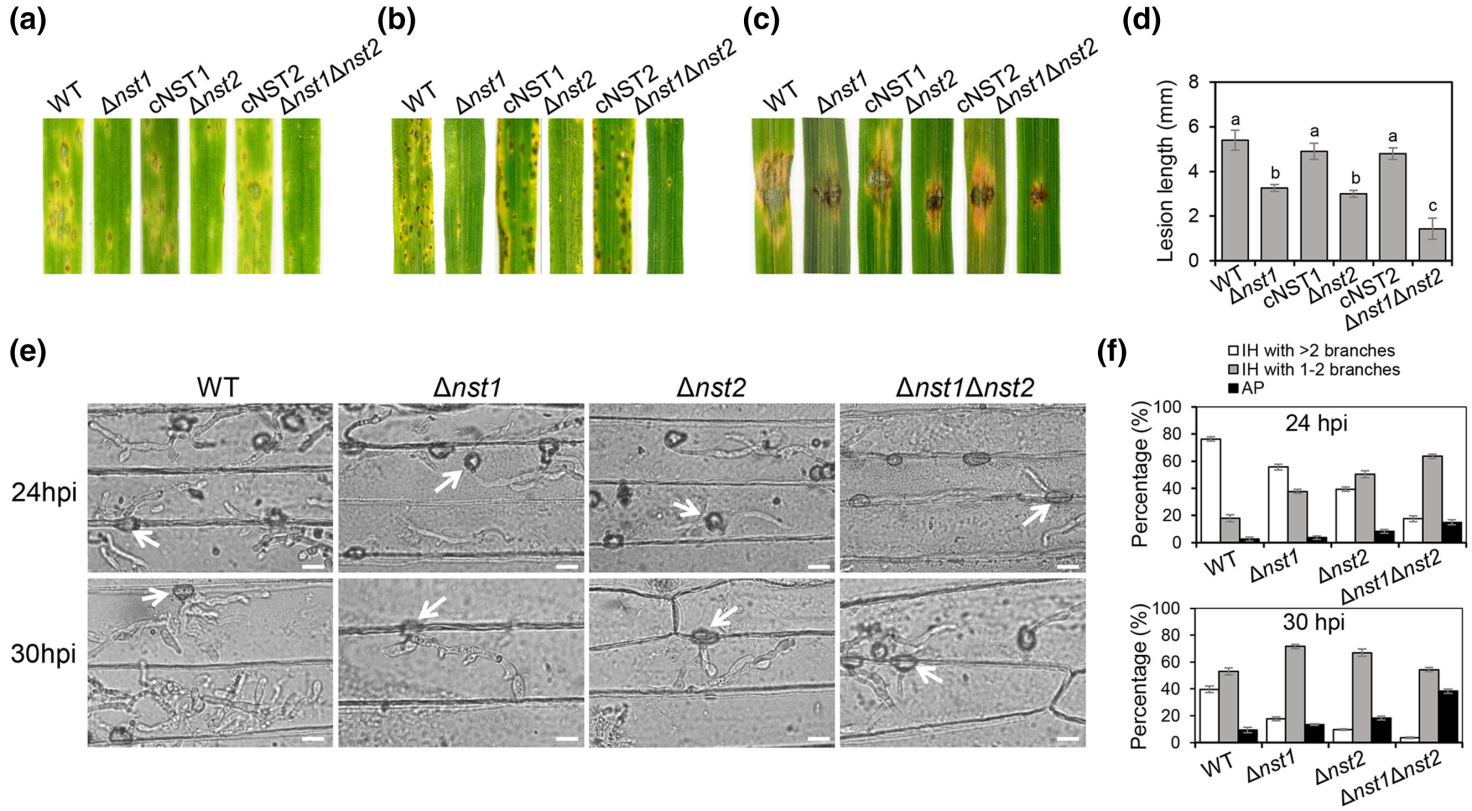
NSTs are required for full virulence of *Magnaporthe oryzae*. (a) Barley leaves were inoculated by spraying with conidial suspensions of wild type (WT), Δ*nst1*, cNST1, Δ*nst2*, cNST2, and Δ*nst1*Δ*nst2*. (b) Rice seedlings were inoculated by spraying with conidial suspensions of the indicated strains. (c) Scratched rice seedlings were inoculated by conidial suspensions of the indicated strains. (d) Statistical analysis of the length of lesions produced from wounded sites in (c) (one‐way analysis of variance [ANOVA], *p* < 0.05). (e) Invasive growth of WT, Δ*nst1*, Δ*nst2*, and Δ*nst1*Δ*nst2* was observed under a microscope. White arrows indicate appressoria. Bar, 10 μm. (f) Statistical analysis of percentages of appressoria (AP) and branched invasive hyphae (IH) in (e) (one‐way ANOVA, *p* < 0.05). WT, wild‐type strain P131; cNST1, *NST1* complementary strain Δ*nst1/NST1*; cNST2, *NST2* complementary strain Δ*nst2/NST2*; Δ*nst1*, *NST1* deletion mutant; Δ*nst2*, *NST2* deletion mutant; Δ*nst1*Δ*nst2*, *NST1* and *NST2* double‐deletion mutant. hpi, hours postinoculation.

Given that *NST1* and *NST2* are highly expressed in appressoria and infection hyphae, we wondered if they are involved in pathogenicity. We inoculated barley and rice seedlings with different strains by spraying with a conidial suspension. Compared with WT, both barley and rice leaves inoculated with Δ*nst1*, Δ*nst2*, and Δ*nst1*Δ*nst2* exhibited fewer and smaller lesions (Figure 6a,b). When inoculating mycelial blocks on wounded rice leaves, we found that lesions of the WT‐infected leaves were significantly larger than those produced by Δ*nst1* and Δ*nst2*. Lesions caused by Δ*nst1*Δ*nst2* showed no obvious expansion (Figure 6c,d), suggesting a redundant function of *NST1* and *NST2* in virulence.

Infection process observations were performed by inoculating a conidial suspension of the above strains on barley epidermis cells or rice leaf sheath. On barley epidermis cells at 24 hpi, almost 40% of WT appressoria formed invasive hyphae containing more than two branches, whereas the percentages of Δ*nst1*, Δ*nst2*, and Δ*nst1*Δ*nst2* were around 18%, 8%, and 2%. Nearly 40% of the Δ*nst1*Δ*nst2* appressoria failed to infect barley cells, but in WT, Δ*nst1*, and Δ*nst2*, it was 6%, 10%, and 15%, respectively. At 3 hpi, nearly 80% of the WT appressoria developed more than two branches, while in Δ*nst1*, Δ*nst2*, and Δ*nst1*Δ*nst2*, it was around 50%, 45%, and 20%, respectively. The majority of the invasive hyphae of Δ*nst1*Δ*nst2* only had one or two branches, and around 15% of the Δ*nst1*Δ*nst2* appressoria did not produce invasive hyphae (Figure 6e,f). In conclusion, both *NST1* and *NST2* are required for full virulence of *M. oryzae*.

### 2.10 NSTs are required for appressorium maturation and adhesion

Because both *NST1* and *NST2* are highly expressed in the mature appressorium stage, we checked if appressorium formation was affected by these two genes. After inoculation for 8 and 12 h on hydrophobic coverslips, the percentages of appressorium formation were calculated. The formation rates of all tested strains were close to 90% at 8 hpi and 95% at 12 hpi. No significant difference was found between mutants and WT (Figure S3a,b), suggesting NSTs are not necessary for appressorium formation.

Infection process observations suggested that *NST1* and *NST2* may be required for appressorial penetration. As appressorial turgor is the key factor for appressorial penetration, we therefore assessed turgor differences among WT and deletion mutants. When treated with 25% polyethylene glycol (PEG) 8000 at 24 hpi, the percentage of collapsed appressoria in WT was less than 10%, significantly less than that in the *nst* deletion mutants. When the PEG 8000 concentration was elevated to 40%, more than 80% of Δ*nst1*Δ*nst2* appressoria collapsed; in comparison, only around 60% of WT appressoria collapsed, less than that of Δ*nst1* or Δ*nst2* (Figure S3c,d). This result shows that *nst* deletion mutants are defective in turgor accumulation for appressorial penetration. Because altered turgor might be related to changes in appressorial mucilage and appressorial adhesion (Rocha et al., 2020), we examined the appressorial mucilage of WT, Δ*nst1*, Δ*nst2*, and Δ*nst1*Δ*nst2*. Scanning electron microscope analysis showed extracellular material, probably mucilage, sparsely accumulated around Δ*nst1* or Δ*nst2* appressoria compared with WT. However, extracellular material was hardly observed around Δ*nst1*Δ*nst2* appressoria (Figure S4a). Furthermore, 96‐well plate assays were conducted to examine appressorial adhesion among the strains. As expected, the appressorial adhesion rate of WT was above 80%, significantly more than the 75.6% of Δ*nst1* and 73.8% of Δ*nst2*. The adhesion rate of Δ*nst1*Δ*nst2* was only around 65% (Figure S4b). Taken together this means that NSTs are required for appressorium maturation and adhesion in *M. oryzae*.

### 2.11 NSTs are important for glycogen utilization during appressorium maturation

During appressorium maturation, the stored glycogen in the conidium will be degraded for utilization (Chen et al., 2022; Deng et al., 2009; Wang et al., 2005). As sugar transporters, *NST* genes might affect glycogen utilization.  Based on this assumption, a KI/I_2_ staining assay was performed to detect glycogen status during appressorium maturation. The percentages of conidia of WT or Δ*nst2* containing glycogen were significantly more than those of Δ*nst1* or Δ*nst1*Δ*nst2* at 8 hpi (Figure S5a,b). At 12 or 24 hpi, the percentage of stained appressoria in WT was significantly less than those for single‐ or double‐deletion mutants, especially in Δ*nst1*Δ*nst2* (Figure S5a,c). These data indicate that *NST*s, particularly *NST1*, play an important role in glycogen utilization during appressorium maturation.

### 2.12 NSTs are involved in protecting against host defence response

**FIGURE 7:**
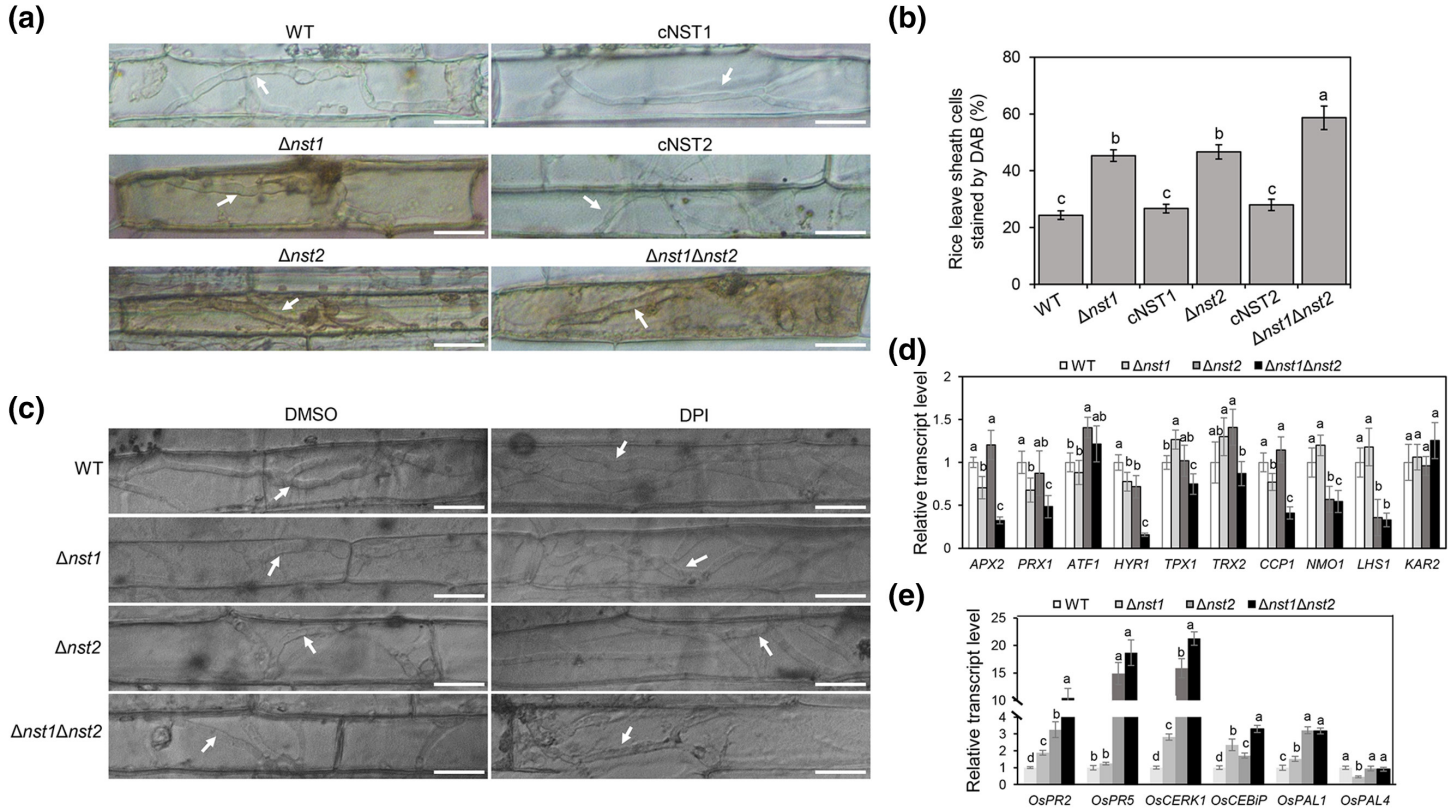
NSTs are important for suppressing host oxidative stress. (a) Host cellular reactive oxygen species (ROS) stained by 3,3′‐diaminobenzidine (DAB) in rice leaf sheaths infected by different strains at 30 h postinoculation (hpi). White arrows indicate invasive hyphae. Bar, 20 μm. (b) Statistical analysis of percentages of infected rice leaf sheath cells stained by DAB (one‐way analysis of variance [ANOVA], *p* < 0.05). (c) Observation of invasive growth at 36 hpi when treated with diphenyleneiodonium (DPI). Dimethyl sulphoxide (DMSO) treatment was a control used to dissolve DPI. Bar, 20 μm. (d) Statistical analysis of the relative expression of ROS detoxification‐related genes in wild type (WT), Δ*nst1*, Δ*nst2*, and Δ*nst1*Δ*nst2* (one‐way ANOVA, *p* < 0.05). *GAPDH* was used as the reference gene. (e) Statistical analysis of the relative expression of disease resistance‐related genes in rice seedlings inoculated with WT, Δ*nst1*, Δ*nst2*, and Δ*nst1*Δ*nst2* for 48 h (one‐way ANOVA, *p* < 0.05). *OsACTIN* was used as the reference gene. WT, wild‐type strain P131; Δ*nst1*, *NST1* deletion mutant; Δ*nst2*, *NST2* deletion mutant; Δ*nst1*Δ*nst2*, *NST1* and *NST2* double‐deletion mutant.

Because Δ*nst1*, Δ*nst2*, and Δ*nst1*Δ*nst2* are deficient in invasive growth and cell wall integrity, we speculated that during infection the *nst* mutants might encounter host reactive oxygen species (ROS) inhibition. On the basis of this hypothesis, H_2_O_2_ was used to detect sensitivity differences among WT, Δ*nst1*, cNST1, Δ*nst2*, cNST2, and Δ*nst1*Δ*nst2*. As expected, the inhibition ratios of the three mutants were much higher than that of the WT, suggesting that *NST1* and *NST2* play roles in the ROS response (Figure 4a,b). Based on this result, we wondered whether deletion of the *NST* genes might result in ROS accumulation in host cells. A 3,3′‐diaminobenzidine (DAB) staining assay was performed to detect the level of ROS produced in rice leaf sheath cells when inoculated with WT and the different mutant stains. At 30 hpi, only around 25% of WT strain‐infected rice cells were stained. In comparison, the percentages of stained cells inoculated by Δ*nst1* and Δ*nst2* were 45% and 47%, and, strikingly, more than 59% of rice cells infected by Δ*nst1*Δ*nst2* were stained by DAB. The percentages of stained cNST1‐ and cNST2‐inoculated rice leaf sheath cells were 27% and 28%, respectively (Figure 7a,b). We assumed limited invasive growth might be caused by defect in scavenging host ROS in the *nst* deletion mutants. We therefore used diphenyleneiodonium (DPI) to inhibit nicotinamide adenine dinucleotide phosphate (NADPH) oxidase activity, which is necessary for host ROS generation (Liu et al., 2021). Compared with the control dimethylsulphoxide (DMSO) treatment, when treated with 0.5 μM DPI, growth of invasive hyphae of Δ*nst1*, Δ*nst2*, and Δ*nst1*Δ*nst2* partially recovered to the WT level at 36 hpi (Figure 7c). These observations suggested that NSTs are involved in preventing or scavenging ROS and the virulence defect of the *nst* deletion mutants may be partly due to the accumulation of host ROS.

Ten reported ROS degradation‐related genes (Guo et al., 2010; Huang et al., 2011; Ren et al., 2022; Yi et al., 2009) were picked to examine their expression among WT and deletion mutants. As expected, the relative expression levels of *HYR1*, *APX2*, *PRX1*, *CCP1*, *NMO1*, and *LHS1* in Δ*nst1*Δ*nst2* were less than half of that in the WT. *TPX1* and *TRX2* showed the lowest expression level in Δ*nst1*Δ*nst2*, although no significant difference of *TRX2* expression was found compared to the WT. *APX2*, *PRX1*, *HYR1*, and *CCP1* exhibited down‐regulated expression in Δ*nst1* compared with that in the WT. *HYR1*, *NMO1*, and *LHS1* showed lower expression levels in Δ*nst2* (Figure 7d). Moreover, expression levels of several rice disease resistance‐related genes were measured in rice leaves inoculated by the four strains. As expected, apart from *OsPAL4*, the other five genes, especially *OsPR2*, *OsPR5*, and *OsCERK1*, showed significantly up‐regulated expression in the deletion mutants compared with those in the WT. The elevated expression levels of *OsPR2*, *OsPR5*, and *OsCERK1* were more obvious in Δ*nst1*Δ*nst2*, which indicates a stronger host defence (Figure 7e). Thus, NSTs play important roles in ROS elimination and the host defence response.

### 2.13 Deletion of *NST*s resulted in reduction of cell wall glycoproteins and chitin exposure

**FIGURE 8:**
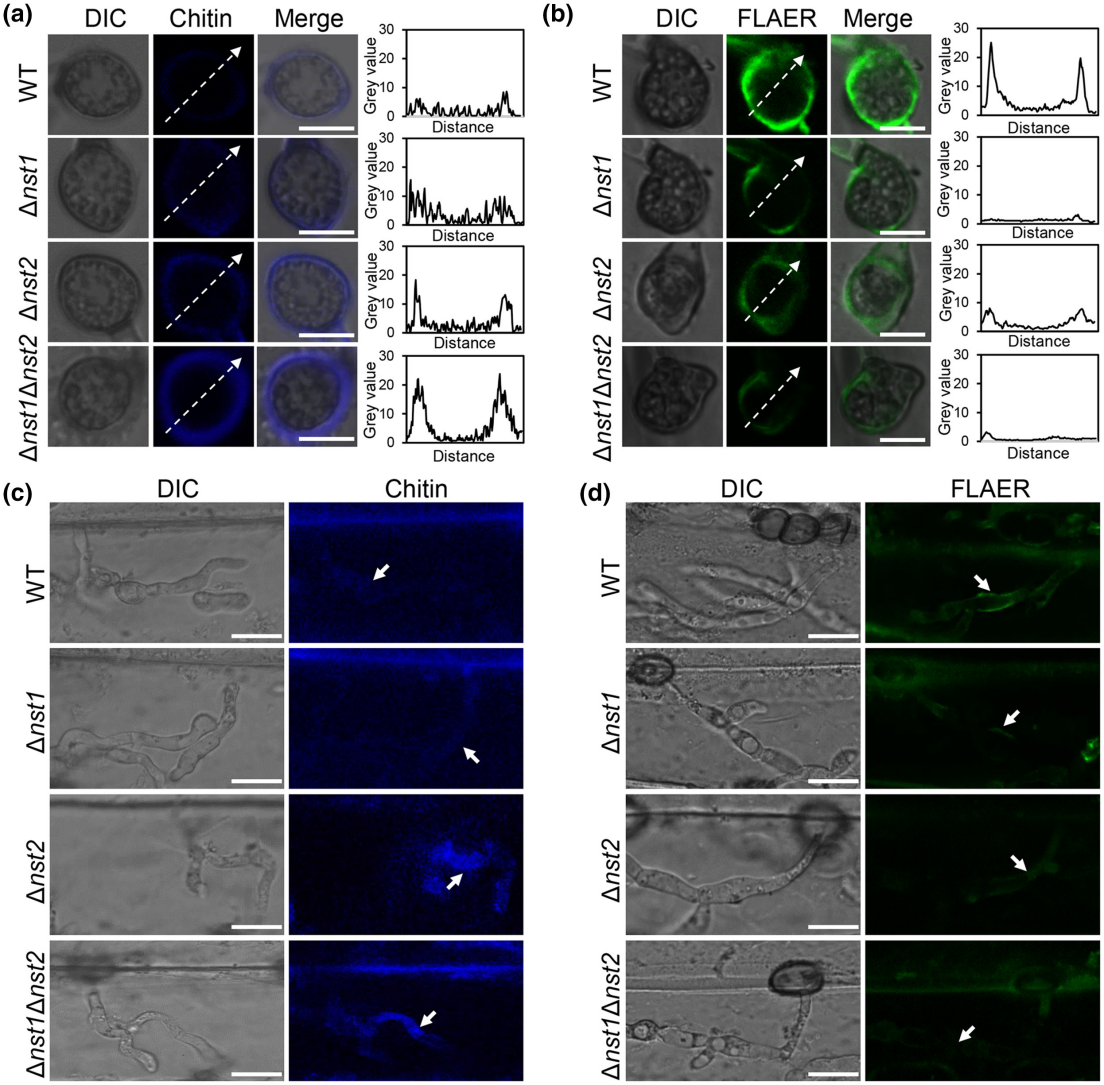
Cell wall structures of *nst* mutants are changed. (a) Detection of chitin in the appressoria of wild type (WT), Δ*nst1*, Δ*nst2*, and Δ*nst1*Δ*nst2* by staining with wheat germ agglutinin (WGA) Alexa Fluor 350. Line‐scan graph analysis shows variation of grey value. Bar, 5 μm. (b) Detection of GPI‐anchored proteins in the appressoria of indicated strains by staining with FLAER. Line‐scan graph analysis shows variation of grey value. Bar, 5 μm. (c) Detection of chitin in infectious hyphae of WT, Δ*nst1*, Δ*nst2*, and Δ*nst1*Δ*nst2*. White arrows show infectious hyphae (IH). Bar, 10 μm. (d) Detection of GPI‐anchored proteins in infectious hyphae of P131, Δ*nst1*, Δ*nst2*, and Δ*nst1*Δ*nst2*. White arrows show IH. Bar, 10 μm.

Because chitin and glycosylphosphatidylinositol (GPI)‐anchored protein are two constituents of the *M. oryzae* cell wall, and GPI‐anchored protein has been shown to shield chitin from host recognition (Liu et al., 2020a), we speculated that deletion of *NST*s would influence chitin exposure or GPI‐anchored protein distribution. To test this, first, trypan blue was applied to stain barley leaf cells inoculated with WT, Δ*nst1*, Δ*nst2*, and Δ*nst1*Δ*nst2* for 30 h, and compared with negative (water‐inoculated leaves) and positive (WT‐inoculated leaves for 5 days) controls. Compared with the positive control, leaves with no inoculation or inoculated with the *nst* mutants could be hardly stained with trypan blue (Figure S6a,b), indicating that the plasma membrane of the host plant during the early stage of fungal invasive growth was still intact. The, wheat germ agglutinin (WGA) Alexa Fluor 350 and FLAER were used to detect chitin and GPI‐anchored proteins, respectively. At 12 hpi, blue fluorescence was hardly observed in appressoria of the WT, but was clearly detectable in *nst* deletion mutants, especially in the double‐deletion mutant (Figure 8a). Conversely, stronger green fluorescence was found in appressoria of the WT, but not in single *nst* mutants, with the weakest fluorescence signal distributed in Δ*nst1*Δ*nst2* appressoria (Figure 8b). At 24 hpi, compared with the WT, much brighter blue fluorescence was present in the infectious hyphae of the two single *nst* deletion mutants and the Δ*nst1*Δ*nst2* double mutant, suggesting clear exposure of chitin in these mutants (Figure 8c). As expected, a stronger green fluorescence signal was observed in the WT infectious hyphae while no obvious signal was detected in the *nst* mutants, suggesting a lack of GPI anchor proteins in the mutants (Figure 8d). This evidence suggests that NSTs affect the distribution of outer layer cell wall glycoproteins, which may protect chitin from host recognition together with above mentioned β‐glucans.

## 3 DISCUSSION

In this study, two putative nucleotide sugar transporters, NST1 and NST2, were identified in *M. oryzae*. We showed that these two NSTs have common and distinct roles in sugar transportation, they are both required for cell wall polysaccharides biogenesis, and therefore they play key roles in cell wall integrity and stress response. We found that NST1 and NST2 are important for hyphal growth and conidial production, and they are also required for full virulence by regulating appressorium maturation, adhesion, and intracellular invasive growth. Moreover, the NSTs are important for *M. oryzae* to keep an intact cell wall structure to avoid recognition by the host immune system. Our results clearly indicate the common and slightly distinct functions of *NST1* and *NST2* in the sugar transport, cell wall integrity, stress response, development, virulence, and immune evasion of *M. oryzae*.

Homologues of *NST1* and *NST2* in *C. neoformans* have been shown to exhibit UDP‐xylose transportation activity and are required for xylose incorporation into the cell wall capsule (Li et al., 2018a). In *Arabidopsis*, triple deletion of *NST1*, *NST2*, and *NST3* resulted in cell wall xylose deposition and xylan biosynthesis. However, in this study, xylose was not identified in the cell wall of the WT and *nst* mutants using GC–MS with xylose standard as a control. We infer that the content of xylose in *M. oryzae* was too low to be extracted, but is abundant in the plant cell wall and the *C. neoformans* polysaccharide capsule (Li et al., 2018a). Because of the structural similarity between xylose and other monosaccharides, we tested to see if the content of other sugars was affected by NSTs. Interestingly, we found that *NST1* might be responsible for the transportation of mannose, glucose, and fructose, while NST2 may be mainly involved in the transportation of sucrose and fructose.

As one of the main cell wall structures, mannoproteins are required for cell wall integrity and fungal virulence (Liu et al., 2020a). Mannose transportation deficiency may cause defects in the biosynthesis of cell wall mannoproteins, leading to abnormal cell wall morphology and reduced pathogenicity. Glucans, polymers of glucose, mainly β‐1,3‐glucan, α‐1,6‐glucan, and β‐1,6‐glucan, are another key component of the *M*. *oryzae* cell wall. According to the GC–MS result, the relative contents of glucose in Δ*nst1* and Δ*nst1*Δ*nst2* (but not in Δ*nst2*) were significantly less than those in the WT. The sugar transportation assay confirmed that *NST1*, but not *NST2*, is responsible for glucose transportation. Thus, it can be concluded that the synthesis of glucans and mannoproteins is affected in *nst* deletion mutants, leading to the defects in cell wall structure.

As the outermost structure of the fungal cell, the cell wall is in contact with environmental agents and host plants. Mannoproteins in the outer layer of the cell wall have been reported to play important roles in the interaction between pathogens and host (Liu et al., 2020a). To be functional, cell wall mannoproteins usually need to be modified by a GPI anchor. During infection, mature appressoria produce penetration pegs with high turgor pressure when in contact with plant cells. The primary IH that emerge from the penetration pegs develop to bulbous IH for further infection (Hu et al., 2022; Liu et al., 2021; Qian et al., 2022). In this study, we detected more GPI‐anchored proteins (GPI‐AP) distributed in the appressoria and IH in the WT than in the *nst* deletion mutants, especially in Δ*nst1*Δ*nst2*. Because some extracellular hydrolytic enzymes or other proteins involved in the cell wall structure were identified to be GPI‐AP, the GPI‐AP can facilitate plant invasion of fungal pathogens. This may explain why the deletion mutants are defective in host penetration.

During the interaction between plants and pathogens, host plants can activate the immune response by recognizing the fungal PAMPs (Li et al., 2020b; Liu et al., 2020b; Wang et al., 2022). As a component of the *M. oryzae* cell wall, chitin is considered to be a canonical PAMP. However, fungal pathogens have evolved to protect chitin from being recognized by host plants. Our results suggest that chitin was easily detected in the appressorium and IH of Δ*nst1*Δ*nst2*, while less chitin was distributed in the cell wall of Δ*nst1* or Δ*nst2*. Compared with the WT, deletion of *NST*s led to failure in protection of chitin from being recognized by plants, and thus resulted in PTI activation. NSTs are therefore fungal virulence factors involved in immune evasion. Fungal pathogens also secrete effector proteins to suppress plant immunity and facilitate tissue invasion. Two distinct secretion systems are used by *M*. *oryzae* to deliver effectors during plant infection. Cytoplasmic effectors, such as Pwl2, Bas1, and AvrPiz‐t, preferentially accumulate in the biotrophic interfacial complex (BIC). In contrast, apoplastic effectors, such as Bas4, Slp1, and Avr1‐CO39, are secreted into the extra‐invasive hyphal membrane (EIHM), following the conventional ER‐Golgi secretory pathway (Giraldo et al., 2013; Zhang & Xu, 2014). Because NSTs transport sugars from the cytosol to the cell surfaces via the ER‐Golgi secretory pathway, we wondered if apoplastic effector secretion was affected by NSTs in *M. oryzae*. *MoSlp1* fused with *GFP* was separately transformed into WT, Δ*nst1*, Δ*nst2*, and Δ*nst1*Δ*nst2*. However, obvious fluorescence signals within the EIHM compartment enclosing the IH were observed in all strains expressing GFP‐MoSlp1 (Figure S7), suggesting that NSTs are unnecessary for apoplastic effector secretion.

The loss of NSTs results in pleiotropic phenotypes, including defects in hyphal growth, conidial production, stress response, appressorium‐mediated penetration, as well as invasive growth in host cell. We think most of the phenotypic defects can be attributed to the damage to cell wall structures in the deletion mutants. Cell wall biosynthesis is responsible for cell shape and morphogenesis during cell growth in yeast and filamentous fungi (Cabib et al., 2001; Gow et al., 2017; Orlean, 2012). As a multipolymer structure, the fungal cell wall balances rigidity and strength to combat internal turgor pressure, and it also possesses enough plasticity to deposit new material at active zones for growth (Cabib et al., 2001; Gow et al., 2017). Fungal cell wall biogenesis has also been reported to be important for sporulation, for example the cell wall integrity pathway is required for yeast sporulation (Piccirillo et al., 2015). Cell wall integrity is also important for appressorial turgor accumulation and appressorial adhesion, which are essential for host penetration (Rocha et al., 2020; Ryder et al., 2022). Together with the above‐mentioned roles of NSTs in invasive growth and stress response, we conclude that pleiotropic phenotypes of the *nst* mutants resulted from defects in cell wall integrity.

Taken together, our results demonstrate that two novel sugar transporters are required for infection of *M. oryzae*. In the future, it will be interesting to identify more NSTs and reveal their roles in sugar transport in plant‐pathogenic fungi.

## 4 EXPERIMENTAL PROCEDURES

### 4.1 Strains and culture conditions

The strain P131 was used as the *M. oryzae* WT (Peng & Shishiyama, 1988; Xue et al., 2012). All strains listed in Table S1 were cultured on OTA plates at 28°C. The colony diameters on the OTA plates were measured at 5 days postinoculation (dpi). Conidia from 7‐day‐old colonies cultured on OTA plates were harvested for testing. Strains cultured in liquid CM at 28°C were used for DNA and RNA extraction.

To test the fungal response to environmental stresses, mycelial blocks were inoculated onto CM plates supplemented with 0.2 mg/ml CR (Sigma‐Aldrich), 0.1 mg/ml CFW (Sigma‐Aldrich), 0.005% SDS, 0.7 M NaCl, 1 M sorbitol, or 10 mM H_2_O_2_. The colony diameters were measured at 5 dpi. To detect fungal utilization of carbon or sugar sources, mycelial blocks were inoculated onto MM plates supplemented with 50 mM glucose, sucrose, galactose, acetate, ethanol, or glycerol. The colony diameters were measured at 5 dpi.

### 4.2 Reverse transcription‐qPCR analysis

To evaluate the expression profile of *NST1* and *NST2*, conidia produced from OTA plates, appressoria at 3 and 12 hpi on Teflon films, and invasive hyphae at 18, 24, and 48 hpi in barley epidermis were harvested to extract total RNA for preparing cDNA templates by reverse transcription. To test the relative expression of ROS degradation‐related genes, the total RNA of strains cultured in CM for 48 h was extracted and reverse transcribed to generate cDNA. To evaluate the expression of rice resistance‐related genes, the total RNA of rice (cv. LTH) seedlings inoculated by conidial spraying was extracted. qPCR was performed using an SYBR Green PCR Master Mix kit (Takara) on an ABI 7500 real‐time PCR system (Applied Biosystems). The *M. oryzae GAPDH* gene and the rice *ACTIN* gene were used as the fungus and rice reference genes, respectively.

### 4.3 Gene disruption and complementation

The split‐PCR strategy was used for gene disruption as previously described (Goswami, 2012). To delete *NST1* or *NST2*, hygromycin (*HYG*) was used as a bridge gene to generate recombinant fragments. Transformants were screened using 250 μg/ml hygromycin B (Roche Diagnostics). The left and right flanking fragment of *NST* genes from transformants were confirmed by PCR using the LBCK/HPT‐up and RBCK/HPT‐down primer pairs (Table S3). Gene deletion was further confirmed by PCR of an internal fragment of *NST1* or *NST2*. For double deletion of *NST1* and *NST2*, neomycin (*NEO*) was selected as a bridge gene to generate recombinant fragments of *NST2*, followed by transformation into Δ*nst1*. Transformants were selected by 400 μg/ml G418 (Amresco). The flanking segments of *NST2* from transformants were confirmed by PCR using the NST2LBCK/NEO‐up and NST2RBCK/NEO‐down primer pairs (Table S3). To generate the complementary strains, a vector containing a 1.5‐kb promoter region, the *NST1* or *NST2* gene coding region, and the adjacent 0.5 kb downstream region was separately transformed into the Δ*nst1* or Δ*nst2* strains. CM plates supplemented with 400 μg/ml G418 were used to select the complementation transformants followed by PCR and phenotype confirmation.

### 4.4 Observation of conidiophores and conidia

Mycelia of P131 and the deletion mutants grown on OTA for 5 days was scraped into a sterile tube containing two small steel balls, then 1 ml of sterile double‐distilled water was added. The mycelium was broken up with a vibrator for 30 s and transferred to a new thicker OTA plate, and evenly smeared on the surface of the medium. The mixture was dried and cultured at 28°C for 36 h until new hyphae grew on the surface. The hyphae were stirred and washed with sterile water, then dried. A sterilized blade was used to cut long pieces of mycelia at the edge of the colony. The mycelial pieces were placed close to each other on a coverslide. It was then incubated at 28°C under light and photographs were taken under a microscope after 18 h.

### 4.5 Appressorium formation and turgor measurement

Conidial suspension drops (2 × 10^5^ spores/ml) were inoculated on a microscope cover glass (12540A; ThermoFisher) and incubated at 28°C under darkness for 8 and 12 h before observation. The appressorium formation rate was defined as the number of appressoria produced in every 100 conidia. For appressorial turgor measurement, appressoria formed on the microscope cover glass were treated by 25% and 40% PEG 8000 for 5 min before observation. The turgor generation was estimated by counting the number of collapsed appressoria in every 100 appressoria under the microscope.

### 4.6 Appressorial adhesion assay

The appressorial adhesion assay was performed as previously described (O'Toole et al., 1999; Rocha et al., 2020; Skamnioti & Gurr, 2007). One hundred microlitres of conidial suspension at a concentration of 10^4^ spores/ml was inoculated in the wells of 96‐well ELISA plates (ThermoFisher) at 28°C in darkness. At 24 hpi, the plates were washed three times by tapping out the contents of the wells and submerging them in sterile distilled water. After air‐drying, 100 μl of 0.05% (wt/vol) crystal violet (BS941; Biosharp) was added to each well and the plates were inoculated for 20 min at room temperature. The plates were washed again three times with sterile distilled water and air dried. One hundred microlitres of 98% ethanol was added to each well followed by gentle shaking for 15 min. The absorbance at 570 nm was measured by a multifunctional microplate detector (Spark; Tecan). The appressorial adhesion values were found by comparison with controls, which were air‐dried but not washed at 24 hpi. These assays were conducted with three biological replicates.

### 4.7 Staining assays

The conidia of different strains were collected by centrifugation and stained with 10 μg/ml CFW solution (Sigma‐Aldrich). The stained conidia were observed and photographed under a fluorescence microscope (Ni90; Nikon), and the proportion of conidia with different numbers of septa was measured. The coverslip was inserted to the edge of the colony on the OTA plate. The hyphal tips grown for 12 h on the coverslip were stained with CFW solution for 10 min. The samples were washed twice with phosphate‐buffered saline before being observed and photographed under the fluorescence microscope.

For the glycogen staining assay, the conidia of the tested strains were diluted to 10^5^ spores/ml. The conidia were inoculated on the hydrophobic plastic cover glass, then kept moist in the culture dish and cultured in the dark. Samples were taken at different time points (0, 3, 8, 12, 24 h) for staining with KI/I_2_ solution (60 mg/ml KI, 10 mg/ml I_2_) for 10 min. Photographs were taken under the microscope.

For the ROS staining assay, barley leaves infected by the tested strains at 30 hpi were stained with 1 mg/ml DAB (Sigma‐Aldrich) solution (pH 3.8) for 12 h, then destained with ethanol/acetic acid solution (94:4) for 4 h on a shaker. Then, 0.5 μM diphenyleneiodonium (DPI) was added to the conidial droplet to inhibit ROS. The whole leaf was observed and photographed under a fluorescence microscope (Ni90; Nikon).

For the chitin staining assay, cell wall chitin of fungal strains was stained with 10 mg/ml wheat germ agglutinin (WGA) Alexa Fluor 350 conjugate (Invitrogen) for 30 min before observation. To detect the GPI‐anchored proteins, appressoria and infectious hyphae of *M. oryzae* were stained with 50 nM fluorochrome (Alexa 488)‐labelled inactivated aerolysin (FLAER) (Pinewood Scientific Services) for 30 min and then observed using a microscope (TCS SP8; Leica) (Liu, et al., 2020a).

For the trypan blue staining assay, barley epidermis inoculated with the indicated strains was soaked in 0.4% (wt/vol) trypan blue solution for 10 min. The stained samples were then rinsed with sterile water three times to remove any remaining trypan blue before observation under a fluorescence microscope (Ni90; Nikon).

For the cell wall composition staining assay, 10 μg/ml CFW and 10 μg/ml ConA Alexa Fluor 350 conjugate (C11254; ThermoFisher) were used to stain vegetative hyphae for 5 and 30 min, respectively. The stained strains were observed using a confocal microscope (TCS SP8; Leica).

### 4.8 Virulence test assays

Rice seedlings (cv. LTH) grown for 4 weeks and barley (cv. E9) grown for 1 week were used for virulence tests. For spray inoculation, the conidial concentration was adjusted to 2 × 10^4^ conidia/ml in 0.025% Tween 20 to spray the barley and 1.5 × 10^5^ conidia/ml to spray the rice leaves. The leaves were incubated under 100% humidity at 28°C with 12 h of darkness and 12 h of light per day. After 4 days of barley inoculation and 5 days of rice inoculation, the inoculated leaves were observed and photographed. For infection process observation, drops of conidial suspension (2 × 10^5^) were inoculated onto barley leaves. The leaf epidermis was collected and observed at 24 and 30 hpi under a fluorescence microscope (Ni90; Nikon).

### 4.9 Subcellular localization observation

The gene coding regions of *NST1* and *NST2* linked with their native promoter were amplified and ligated to the 5′ end of the *GFP* gene in the vector pGTN (Tables S2 and S3; Liu et al., 2018). The resultant vectors pGTN‐NST1 or pGTN‐NST2 were cotransformed with pKNRR‐HDEL into Δ*nst1* or Δ*nst2*, respectively. Transformants at different developmental stages were used to observe subcellular localization under a confocal laser scanning microscope (Leica). For detection of Slp1 localization, the pGTN‐MoSlp1 vector was generated and separately transformed into WT, Δ*nst1*, Δ*nst2*, and Δ*nst1*Δ*nst2*. The resulting transformants were used to inoculate barley leaves. At 28 hpi, green fluorescence was observed under a confocal laser scanning microscope.

### 4.10 Scanning electron microscope analysis

Barley leaves were inoculated with conidial suspension (10^6^) for 12 h at 25°C under 100% humidity with darkness. Samples were analysed by a LEO 1530 scanning electron microscope (Zeiss).

### 4.11 Extraction of soluble cell wall saccharides

Cell walls were isolated according to a previous method (Feiz et al., 2006; Liu, et al., 2020a). Equal amounts (0.5 g) of mycelia of WT, Δ*nst1*, Δ*nst2*, and Δ*nst1*Δ*nst2* were collected from liquid CM cultures and rinsed with 5 mM acetate buffer (pH 4.6). The mycelia were stirred in a blender with 100 ml of a solution containing 0.4 M sucrose and 5 mM acetate buffer for 5 min at 4°C. The mixture was then centrifuged at 2000 × *g* for 15 min at 4°C and the precipitate was resuspended in a solution containing 0.4 M sucrose and 5 mM acetate buffer. The centrifugation step was repeated twice by sequentially resuspending the precipitate in solutions containing 0.6 M sucrose and 1 M sucrose in 5 mM acetate buffer (pH 4.6). The resulting pellet was considered to be the cell wall fraction.

The cell wall fractions of WT, Δ*nst1*, Δ*nst2*, and Δ*nst1*Δ*nst2* were transferred to a new 2 ml tube then 1.5 ml of 75% methanol (chromatographic grade) was added. The mixture was vortexed thoroughly and incubated in a water bath at 70°C for 15 min, with mixing upside down for three times, followed by centrifugation at 10,000 × *g* for 10 min at room temperature. For each sample, 500 μl of supernatant was transferred into a new 1.5‐ml tube containing 50 μl ribitol (50 μg/ml, as an internal standard). This liquid was vortexed briefly and centrifuged at 10,000 × *g* for 30 s at room temperature. The mixture was frozen in liquid nitrogen then immediately dried in a vacuum freeze‐drier. The resulting freeze‐dried powders were the soluble cell wall saccharides.

### 4.12 Derivatization and GC–MS analysis

The experimental steps of derivatization were conducted according to a previous method (Xu et al., 2021). The freeze‐dried powder was deposited to the tube bottom by centrifugation at 10,000 × *g* for 30 s. Then 50 μl of methoxyamine hydrochloride (15 mg/ml, dissolved in pyridine; Sigma‐Aldrich) was added to each tube and the powder dissolved completely followed by centrifugation at 10,000 × *g* for 2 min at room temperature. The solution was transferred carefully to an autosampler vial with a glass insert and placed in an incubator at 65 °C for 2 h. Fifty microlitres of N‐methyl‐N‐(trimethylsilyl) trifluoroacetamide (M0672; TCI) was added to the autosampler vial and the solution was kept at 65°C for another 2 h. The sample was then subjected to analysis by GC–MS (QP‐2010; Shimadzu).

The GC parameters were set as follows: carrier gas helium (53.6 kPa), gas flow rate 1 ml/min, GC interface temperature 250°C. The GC oven temperature gradient programme was as follows: start at 50°C, hold at 50°C for 1 min, ramp to 150°C at 5°C/min, hold at 150°C for 1 min, ramp to 280°C at 10°C/min, and hold at 280°C for 5 min. The total programme time was 40 min. The MS parameters were set as follows: source temperature 250°C, *m*/*z* range 25–625, scanning speed 0.4 scans/s, and ionization energy 70 eV.

### 4.13 Sugar transportation property analysis

The coding regions of *NST1* and *NST2* were separately inserted into yeast expression vector pDR195. The subsequent vectors were transformed into the hexose transport‐deficient yeast strain EBY.VW4000 by electric shock (2400 V, 5 ms). The resulting transformants were suspended in liquid YNB (PM2070; Coolaber) medium with 2% maltose and Do supplement‐Ura (PM2270; Coolaber). Plasmids were then extracted using a kit (D3376; Omega) and identified by PCR. The positive transformants were cultured to an appropriate OD_600_ (0.5–0.7), followed by centrifugation at 8000 × *g* for 5 min. The pellet was resuspended in 100 mM phosphate‐buffered saline (pH 6.5) and adjusted to concentrations of 10^7^, 10^6^, 10^5^, 10^4^, and 10^3^ cells/ml. Two microlitres of each yeast suspension was dropped onto solid YNB medium containing with Do supplement‐Ura and 2% target sugar. The plates were incubated at 30°C for 48 h before observation.

### 4.14 Statistical analysis

All values represent the mean of at least three biological replicates. Error bars indicate the standard deviation. Statistical comparisons were performed using one‐way analysis of variance (*p* < 0.05) in SPSS 19.0 software (IBM).

## CONFLICT OF INTEREST STATEMENT

The authors declare no conflict of interest exists.

## Supporting information


**Figure S1.** Knockout strategy and confirmation of Δ*nst1*, Δ*nst2*, and Δ*nst1*Δ*nst2*. (a) Deletion diagram of Δ*nst1* or Δ*nst2*. (b) PCR confirmation of two *NST1* deletion mutants. (c) PCR confirmation of two *NST2* deletion mutants. (d) Deletion diagram of Δ*nst1*Δ*nst2*. (e) PCR confirmation of two *NST1* and *NST2* double‐deletion mutants

**Figure S2.** Peak figure of standard fructose, galactose, mannose, glucose, and sucrose. Purple, blue, green, yellow, and red asterisks show the peak positions of ribitol, fructose, galactose, mannose, glucose, and sucrose, respectively. The black asterisk indicates the peak position of ribitol added in every sugar standard sample

**Figure S3.** NSTs are important for functional appressoria formation. (a) Appressoria (AP) formation of P131, Δ*nst1*, Δ*nst2*, and Δ*nst1*Δ*nst2* on the hydrophobic slide at 8 and 12 h postinoculation (hpi). White arrows indicate appressoria. Bar, 20 μm. (b) Statistical analysis of appressoria (AP) formation rate of the indicated strains (one‐way analysis of variance [ANOVA], *p* > 0.05). (c) Observation of collapsed AP when 25% or 40% PEG 8000 added at 24 hpi on the hydrophobic slide. White arrows indicate collapsed appressoria. Bar, 20 μm. (d) Statistical analysis of collapsed AP rate of the four strains when treated by 25% or 40% PEG 8000 (one‐way ANOVA, *p* < 0.05)

**Figure S4.** (a) Scanning electron microscopy observation of extracellular materials of appressoria. Bar, 5 μm. (b) Statistical analysis of appressoria adhesion percentages (one‐way analysis of variance, *p* < 0.05)

**Figure S5.** NSTs play important roles in utilization of glycogen and sugars. (a) KI/I_2_ stained glycogen in conidia (CO) and appressoria (AP) of indicated strains. The strains were cultured on the hydrophobic slide for 0, 3, 8, 12, and 24 h, stained by KI/I_2_, and observed under a fluorescence microscope. Bar, 20 μm. (b) Statistical analysis of percentages of conidia containing glycogen in (a) (one‐way analysis of variance [ANOVA], *p* < 0.05). (c) Statistical analysis of percentages of appressoria containing glycogen in (a) (one‐way ANOVA, *p* < 0.05)

**Figure S6.** Host cell membrane integrity test of barley epidermis cells inoculated by *Magnaporthe oryzae*. (a) Trypan blue staining of barley leaves inoculated by wild type **(**WT), Δ*nst1*, Δ*nst2*, or Δ*nst1*Δ*nst2* for 26 h. Leaves inoculated with water for 26 h and WT for 5 days (5 dpi) were used as the negative and positive controls, respectively. Bar, 10 μm. (b) Statistical analysis of percentages of stained barley cells in (a) (one‐way analysis of variance, *p* < 0.01)

**Figure S7.** NSTs are not required for apoplastic effector secretion. Subcellular localization of GFP‐MoSlp1 expressed in wild type (WT), Δ*nst1*, Δ*nst2*, or Δ*nst1*Δ*nst2* were observed at 28 h postinoculation in barley epidermis cells. Asterisks indicate the appressorium. White arrows indicate the probable extra‐invasive hyphal membrane. Bar, 10 μm

**Table S1.** Fungal strains used in this study

**Table S2.** Plasmids used in this study

**Table S3.** Primers used in this study

## Data Availability

The data that support the findings of this study are available from the corresponding author upon reasonable request.
