# Supplementary material for: Two nucleotide sugar transporters are important for cell wall integrity and full virulence of Magnaporthe oryzae
Source: Mol Plant Pathol. 2023 Feb 12;24(4):374–90. doi: 10.1111/mpp.13304 (PMC10013753; doi:10.1111/mpp.13304)
Supplement: Supplementary file 1 — Figure S1. Knockout strategy and confirmation of Δnst1, Δnst2, and Δnst1Δnst2. (a) Deletion diagram of Δnst1 or Δnst2. (b) PCR confirmation of two NST1 deletion mutants. (c) PCR confirmation of two NST2 deletion mutants. (d) Deletion diagram of Δnst1Δnst2. (e) PCR confirmation of two NST1 and NST2 double‐deletion mutants [file MPP-24-374-s002.pdf]

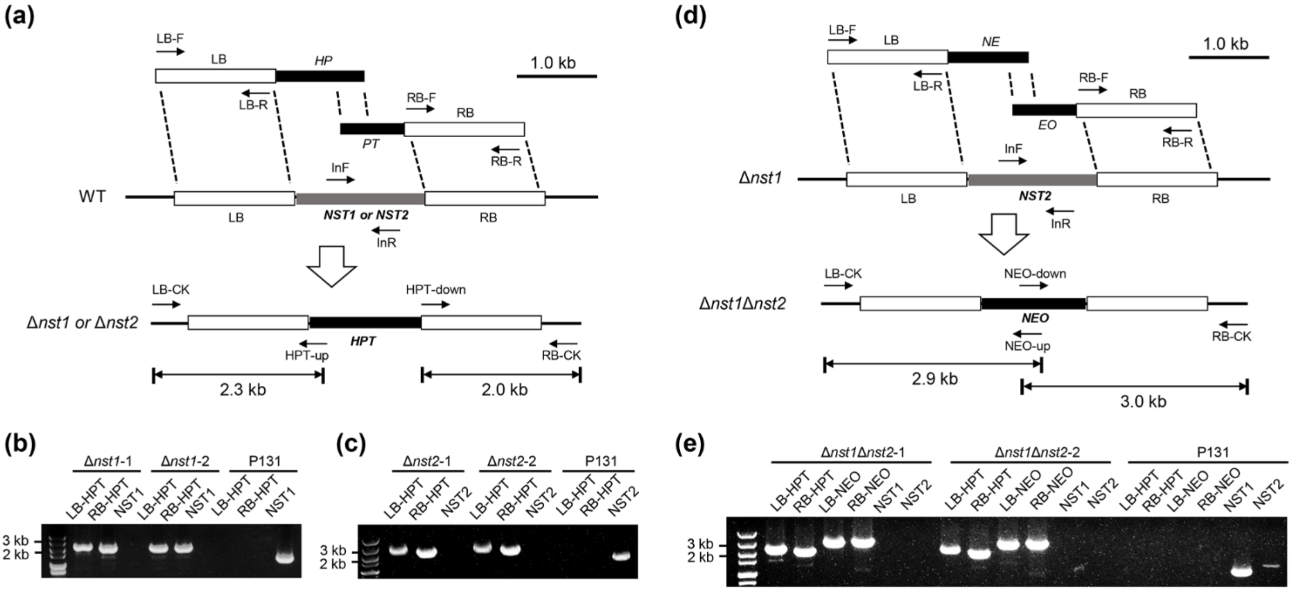

Figure S1. Knock-out strategy and confirmation of Δ*nst1*, Δ*nst2*, and Δ*nst1*Δ*nst2*. (a) Deletion diagram of Δ*nst1* or Δ*nst2*. (b) PCR confirmation of two *NST1* deletion mutants. (c) PCR confirmation of two *NST2* deletion mutants. (d) Deletion diagram of Δ*nst1*Δ*nst2*. (e) PCR confirmation of two *NST1* and *NST2* double deletion mutants.
