# Supplementary material for: Two nucleotide sugar transporters are important for cell wall integrity and full virulence of Magnaporthe oryzae
Source: Mol Plant Pathol. 2023 Feb 12;24(4):374–90. doi: 10.1111/mpp.13304 (PMC10013753; doi:10.1111/mpp.13304)
Supplement: Supplementary file 2 — Figure S2. Peak figure of standard fructose, galactose, mannose, glucose, and sucrose. Purple, blue, green, yellow, and red asterisks show the peak positions of ribitol, fructose, galactose, mannose, glucose, and sucrose, respectively. The black asterisk indicates the peak position of ribitol added in every sugar standard sample [file MPP-24-374-s003.pdf]

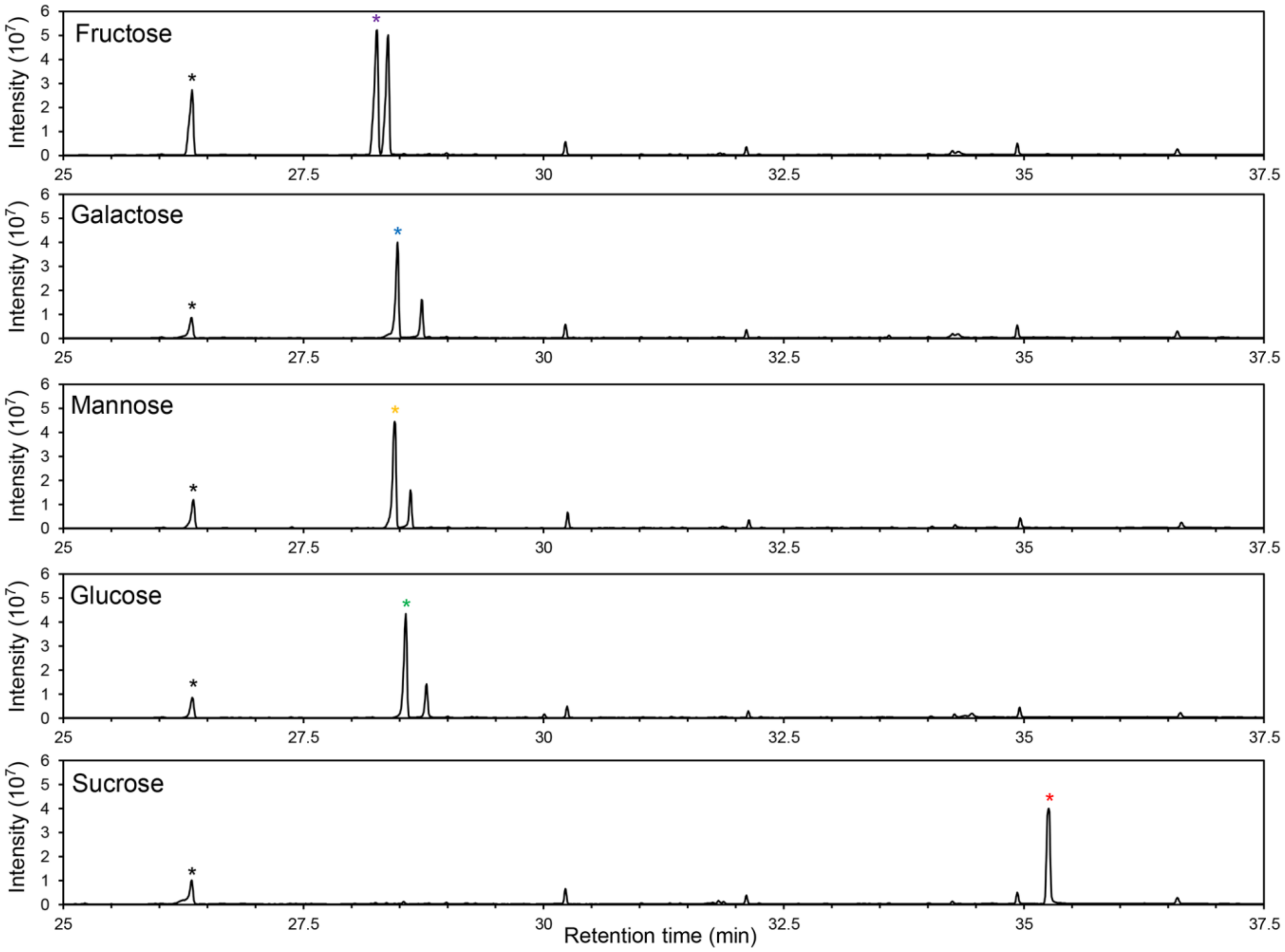

Figure S2. Peak figure of standard fructose, galactose, mannose, glucose, and sucrose. Purple, blue, green, yellow, and red asterisks show the peak position of ribitol, fructose, galactose, mannose, glucose, and sucrose, respectively. The black asterisk indicates the peak position of ribitol added in every sugar standard sample.
