# Supplementary material for: Two nucleotide sugar transporters are important for cell wall integrity and full virulence of Magnaporthe oryzae
Source: Mol Plant Pathol. 2023 Feb 12;24(4):374–90. doi: 10.1111/mpp.13304 (PMC10013753; doi:10.1111/mpp.13304)
Supplement: Supplementary file 3 — Figure S3. NSTs are important for functional appressoria formation. (a) Appressoria (AP) formation of P131, Δnst1, Δnst2, and Δnst1Δnst2 on the hydrophobic slide at 8 and 12 h postinoculation (hpi). White arrows indicate appressoria. Bar, 20 μm. (b) Statistical analysis of appressoria (AP) formation rate of the indicated strains (one‐way analysis of variance [ANOVA], p > 0.05). (c) Observation of collapsed AP when 25% or 40% PEG 8000 added at 24 hpi on the hydrophobic slide. White arrows indicate collapsed appressoria. Bar, 20 μm. (d) Statistical analysis of collapsed AP rate of the four strains when treated by 25% or 40% PEG 8000 (one‐way ANOVA, p < 0.05) [file MPP-24-374-s001.pdf]

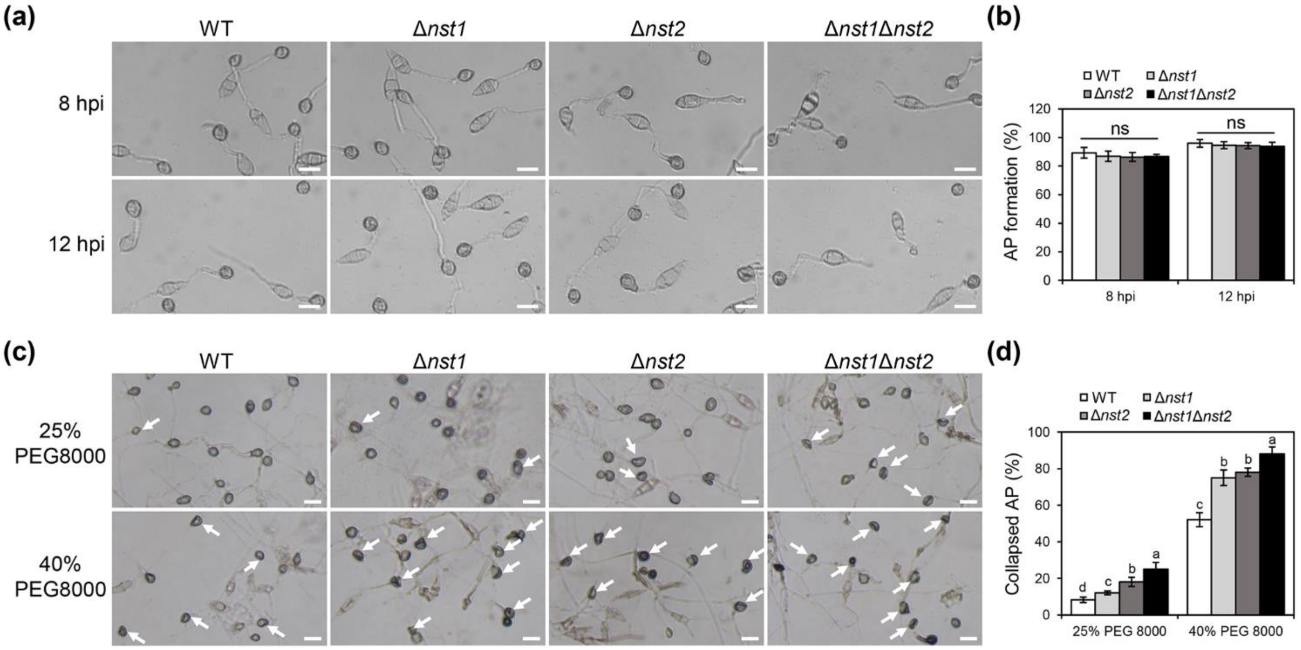

Figure S3. NSTs are important for functional appressoria formation. (a) Appressoria formation of P131, Δ*nst1*, Δ*nst2*, and Δ*nst1*Δ*nst2* on the hydrophobic slide at 8 hpi and 12 hpi. White arrows indicate appressoria. Bar, 20 μm. (b) Statistical analysis of appressoria (AP) formation rate of the indicated strains (one-way AVONA: $P > 0.05$). (c) Observation of collapsed AP when 25% or 40% PEG 8000 added at 24 hpi on the hydrophobic slide. White arrows indicate collapsed appressoria. Bar, 20 μm. (d) Statistical analysis of collapsed AP rate of the four strains when treated by 25% or 40% PEG 8000 (one-way AVONA: $P < 0.05$).
