# Supplementary material for: Two nucleotide sugar transporters are important for cell wall integrity and full virulence of Magnaporthe oryzae
Source: Mol Plant Pathol. 2023 Feb 12;24(4):374–90. doi: 10.1111/mpp.13304 (PMC10013753; doi:10.1111/mpp.13304)
Supplement: Supplementary file 4 — Figure S4. (a) Scanning electron microscopy observation of extracellular materials of appressoria. Bar, 5 μm. (b) Statistical analysis of appressoria adhesion percentages (one‐way analysis of variance, p < 0.05) [file MPP-24-374-s010.pdf]

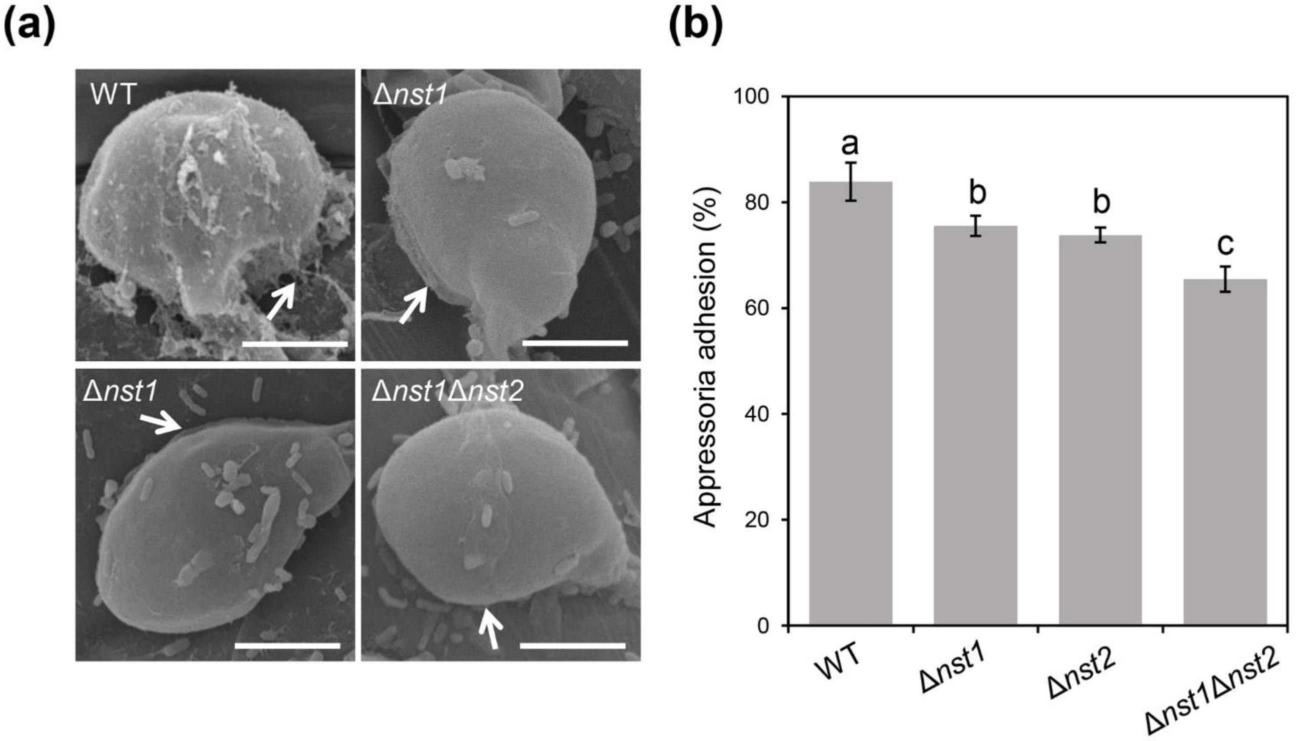

Figure S4. (a) SEM observation of extracellular materials of appressoria. Bar, 5 μm. (b) Statistical analysis of appressoria adhesion percentages (one-way AVONA: $P < 0.05$).
