# Supplementary material for: Two nucleotide sugar transporters are important for cell wall integrity and full virulence of Magnaporthe oryzae
Source: Mol Plant Pathol. 2023 Feb 12;24(4):374–90. doi: 10.1111/mpp.13304 (PMC10013753; doi:10.1111/mpp.13304)
Supplement: Supplementary file 5 — Figure S5. NSTs play important roles in utilization of glycogen and sugars. (a) KI/I2 stained glycogen in conidia (CO) and appressoria (AP) of indicated strains. The strains were cultured on the hydrophobic slide for 0, 3, 8, 12, and 24 h, stained by KI/I2, and observed under a fluorescence microscope. Bar, 20 μm. (b) Statistical analysis of percentages of conidia containing glycogen in (a) (one‐way analysis of variance [ANOVA], p < 0.05). (c) Statistical analysis of percentages of appressoria containing glycogen in (a) (one‐way ANOVA, p < 0.05) [file MPP-24-374-s008.pdf]

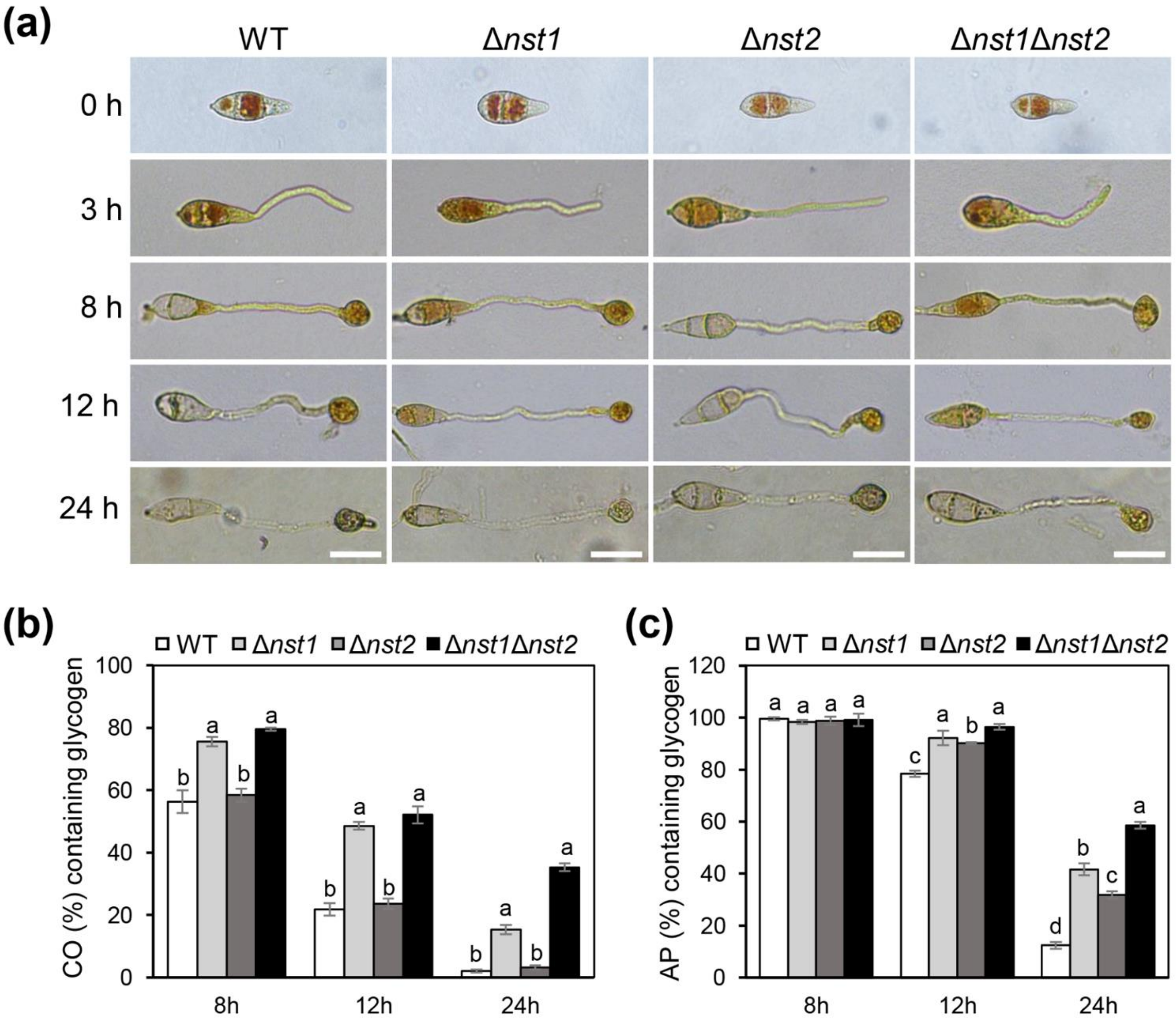

Figure S5. NSTs play important roles in utilization of glycogen and sugars. (a) KI/I_2 stained glycogen in conidia (CO) and appressoria (AP) of indicated strains. The strains were cultured on the hydrophobic slide for 0 h, 3 h, 8 h, 12 h, and 24 h stained by KI/I_2 were observed under a fluorescence microscope. Bar, 20 μm. (b) Statistical analysis of percentages of conidia containing glycogen in (a) (one-way AVONA: $P < 0.05$). (c) Statistical analysis of percentages of appressoria containing glycogen in (a) (one-way AVONA: $P < 0.05$).
