# Supplementary material for: Two nucleotide sugar transporters are important for cell wall integrity and full virulence of Magnaporthe oryzae
Source: Mol Plant Pathol. 2023 Feb 12;24(4):374–90. doi: 10.1111/mpp.13304 (PMC10013753; doi:10.1111/mpp.13304)
Supplement: Supplementary file 6 — Figure S6. Host cell membrane integrity test of barley epidermis cells inoculated by Magnaporthe oryzae. (a) Trypan blue staining of barley leaves inoculated by wild type (WT), Δnst1, Δnst2, or Δnst1Δnst2 for 26 h. Leaves inoculated with water for 26 h and WT for 5 days (5 dpi) were used as the negative and positive controls, respectively. Bar, 10 μm. (b) Statistical analysis of percentages of stained barley cells in (a) (one‐way analysis of variance, p < 0.01) [file MPP-24-374-s004.pdf]

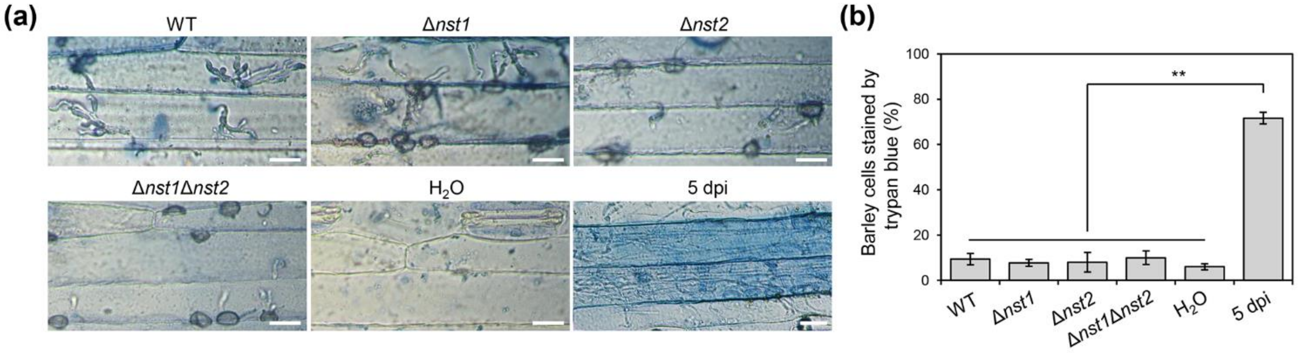

Figure S6. Host cell membrane integrity test of barley epidermis cells inoculated by *M. oryzae*. (a) Trypan blue staining of barley leaves inoculated by WT, Δ*nst1*, Δ*nst2*, or Δ*nst1*Δ*nst2* for 26 hours. Leaves inoculated with water (H_2O) for 26 hours and WT for 5 days (5dpi) were used as the negative and positive control, respectively. Bar, 10 μm. (b) Statistical analysis of percentages of stained barley cells in (a) (one-way AVONA: $P < 0.01$).
