# Supplementary material for: Two nucleotide sugar transporters are important for cell wall integrity and full virulence of Magnaporthe oryzae
Source: Mol Plant Pathol. 2023 Feb 12;24(4):374–90. doi: 10.1111/mpp.13304 (PMC10013753; doi:10.1111/mpp.13304)
Supplement: Supplementary file 7 — Figure S7. NSTs are not required for apoplastic effector secretion. Subcellular localization of GFP‐MoSlp1 expressed in wild type (WT), Δnst1, Δnst2, or Δnst1Δnst2 were observed at 28 h postinoculation in barley epidermis cells. Asterisks indicate the appressorium. White arrows indicate the probable extra‐invasive hyphal membrane. Bar, 10 μm [file MPP-24-374-s006.pdf]

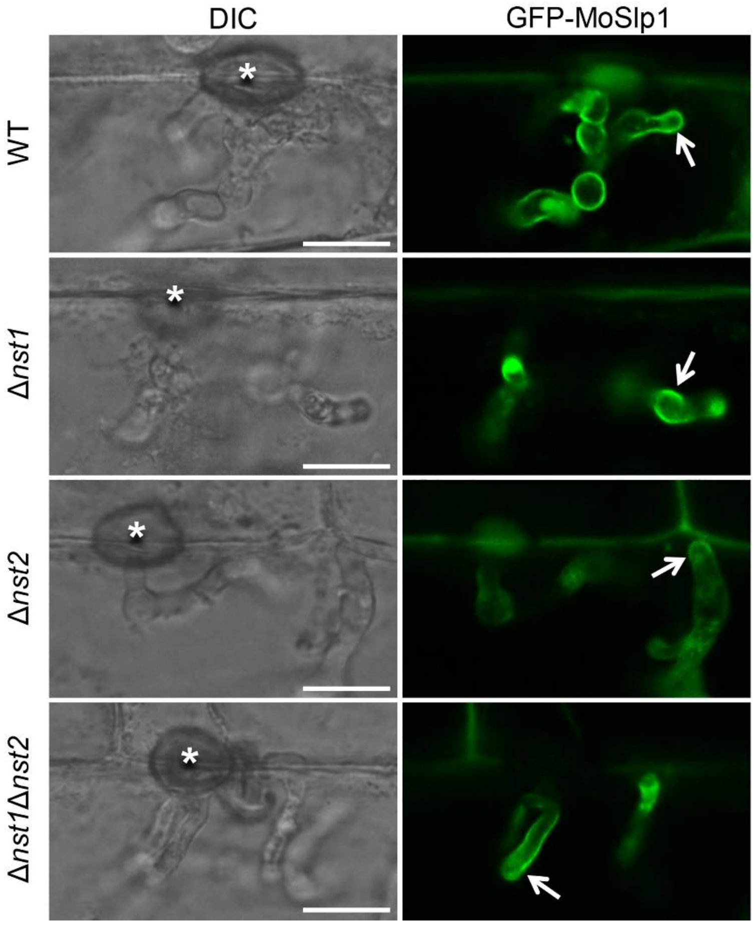

Figure S7. NSTs are not required for apoplastic effector secretion. Subcellular localization of GFP-MoSlp1 expressed in WT, Δ*nst1*, Δ*nst2*, or Δ*nst1*Δ*nst2* were observed at 28 hpi in barley epidermis cells. Asterisks indicate the appressorium. White arrows indicate the probable EIHM. Bar, 10 μm.
