# Supplementary material for: Two nucleotide sugar transporters are important for cell wall integrity and full virulence of Magnaporthe oryzae
Source: Mol Plant Pathol. 2023 Feb 12;24(4):374–90. doi: 10.1111/mpp.13304 (PMC10013753; doi:10.1111/mpp.13304)
Supplement: Supplementary file 8 — Table S1. Fungal strains used in this study [file MPP-24-374-s007.pdf]

Table S1 Fungal strains used in this study.

Strains	Discriptions	References
P131	A wild-type isolate of *M. oryzae*	(Peng and Shishiyama, 1988)
Δ*nst1*	*NST1* deletion mutants of P131	This study
Δ*nst2*	*NST2* deletion mutants of P131	This study
Δ*nst1*Δ*nst2*	*NST2* deletion mutants of Δ*nst1*	This study
cNST1	Complementary strain of Δ*nst1*	This study
cNST2	Complementary strain of Δ*nst2*	This study
NST1GFP-RFPHDEL	Colocalization strain of NST1 and HDEL	This study
NST2GFP-RFPHDEL	Colocalization strain of NST2 and HDEL	This study

Peng, Y.L. & Shishiyama, J. (1988) Temporal sequence of cytological events in rice leaves infected with Pyricularia oryzae. Canadian Journal of Botany, 66, 730-735.
