# Supplementary material for: Two nucleotide sugar transporters are important for cell wall integrity and full virulence of Magnaporthe oryzae
Source: Mol Plant Pathol. 2023 Feb 12;24(4):374–90. doi: 10.1111/mpp.13304 (PMC10013753; doi:10.1111/mpp.13304)
Supplement: Supplementary file 9 — Table S2. Plasmids used in this study [file MPP-24-374-s009.pdf]

Table S2 Plasmids used in this study.

Names	Descriptions
pKN	Vector used to construct complementary vectors, with the *NPTII* gene as a selective marker inserted into pKS^+ (Yang et al., 2010).
pKN-*NST1*	*NST1* complementary vector; *NST1* gene containing 1.5 kb promoter and 0.5 kb terminator regions were amplified and inserted into pKN.
pKN-*NST2*	*NST2* complementary vector; *NST2* gene containing 1.5 kb promoter and 0.5 kb terminator regions were amplified and inserted into pKN.
pGTN	Vector used to construct vectors to express target genes (Yang et al., 2010)
pGTN-*NST1*	Vector for subcellular localization of NST1; 1.5 kb promoter and coding region of *NST1* was cloned into vector pGTN.
pGTN-*NST2*	Vector for subcellular localization of NST2; 1.5 kb promoter and coding region of *NST2* was cloned into vector pGTN.
pKNRR-*HDEL*	ER-localized vector
pDR195-*NST1*	Vector used to express NST1 in yeast strain EBY.VW4000
pDR195-*NST2*	Vector used to express NST2 in yeast strain EBY.VW4000
GFP-MoSlp1	Vector for subcellular localization of MoSlp1

Yang, J., Zhao, X.Y., Sun, J., Kang, Z.S., Ding, S.L., Xu, J.R. et al. (2010) A novel protein Com1 is required for normal conidium morphology and full virulence in Magnaporthe oryzae. Molecular Plant-Microbe Interactions, 23, 112-123.
