# Supplementary material for: Two nucleotide sugar transporters are important for cell wall integrity and full virulence of Magnaporthe oryzae
Source: Mol Plant Pathol. 2023 Feb 12;24(4):374–90. doi: 10.1111/mpp.13304 (PMC10013753; doi:10.1111/mpp.13304)
Supplement: Supplementary file 10 — Table S3. Primers used in this study [file MPP-24-374-s005.pdf]

Table S3 Primers used in this study.

Primers	Sequences (5'–3')
HPT-up	GACAGACGTCGCGGTGAGTT
HPT-down	TCTGGACCGATGGCTGTGTAG
HPT-F1	CTCCGACCTGATGCAGCTCT
HPT-R1	CTCGCTCCAGTCAATGACC
HPT-F	CTTGGCTGGAGCTAGTGGAGGT
HPT-R	CCCGGTCGGCATCTACTCTATTC
NST1LB-CK	TTGCAGTACAGACCTAC
NST1LB-F	TTCTTCTTAGCGTCCTCCCT
NST1LB-R	ACCTCCACTAGCTCCAGCCAAGACTGCTCCCTGATGTTACCC
NST1RB-F	GAATAGAGTAGATGCCGACCGGGCAAAGGGTTACTGTGCGG
NST1RB-R	GTGCAGTTGTTGATCCATTC
NST1RB-CK	TATACGTAGGAGGAGGC
NST1InF	TCGATCAGTGCATGGAT
NST1InR	GACCTTATTGGCAGCCT
NST2LB-CK	CTGCTTCCGTGGTAGTAT
NST2LB-F	GATCTGATAGGCTAGGTG
NST2LR-HPT	ACCTCCACTAGCTCCAGCCAAGGTGCGTCTCCTAGGAATT
NST2LR-NEO	GAAATTGTAAGCGTTAATCTAGGTGCGTCTCCTAGGAATT
NST2RF-HPT	GAATAGAGTAGATGCCGACCGGGTAGATCACTGCAAATCTC
NST2RF-NEO	CCTTCTTGACGAGTTCTTCTGATAGATCACTGCAAATCTC
NST2RB-R	TAGGGATCGTCGTGATGA
NST2RB-CK	GGATGCCAGAGGTGACA
NST2InF	CATCGCGTACGCCTCTT
NST2InR	CTTCAGACTGTAGGTGC
NEO-F1	TTGATACAGCTTCGCAGG
NEO-R1	TATGTCCTGATAGCGGTC
NEO-up	GAGCAAGGTGAGATGACAGGAG
NEO-down	CACCACTCGATCCGTCACCAAC
NEO-F	TCTAGATTAACGCTTACAATTTCC
NEO-R	TCAGAAGAACTCGTCAAGAAGG
NST1GTN-F	TCGAGGTCGACGGTATCGATAAGCTTTGGCCAGAGAACTCGAG
NST1GTN-R	CCAGCACCTCTAGAACTAGTGGATCCTTGAGGCAAACCGACCT
NST2GTN-F	TCGAGGTCGACGGTATCGATAAGCTTAAGTATTCTCGGCAGGCC
NST2GTN-R	CCAGCACCTCTAGAACTAGTGGATCCTCGACGACCAACTATAGT
NST1KN-F	GATATCGAATTCGGGATCCTGGCCAGAGAACTCGAGC
NST1KN-R	GTGGATCCCGAATTCTTCGATCGGATCTTCCG
NST2KN-F	GATATCGAATTCGGGATCCCGCAACGCAGGTGCACCT
NST2KN-R	GTGGATCCCGAATTCTGATATGGACTGGGGATG
NST1-DR195F	CATGGTACCATGAGCACCGAAGACAA
NST1-DR195R	CATCTCGAGCTATTGAGGCAAACCGAC
NST2-DR195F	CATGGTACCATGTCGGCGTCGGGTTTG
NST2-DR195R	CATCTCGAGCTATCGACGACCAACTAT
APX2-qF	CGTCGTCACCGAGTATCTGG
APX2-qR	CCTCATTGCGTTGACGGTTG
PRX1-qF	CTACCAGGACACCACCAACG
PRX1-qR	CCGGGTACGACAGAATGGTC
ATF1-qF	CGGCAAACGGCCTCTTTATG
ATF1-qR	AGGTGACGTCTTGATGGCAG

HYR1-qF	ATGGCTTCCGCTACGACAAT
HYR1-qR	TTGGAGGCCGTGTTGACTAC
TPX1-qF	CTCTGTGAACGGCAAGGAGT
TPX1-qR	ACCAGTTGCTTCAGGGTGAC
TRX2-qF	TTTCAAATCCGGTCGCTTGC
TRX2-qR	TTGTTGCTGATTGCGACGTG
CCP1-qF	CCGTGTACAACGACATTGCC
CCP1-qR	CTCCTTGTCGTAGGTACCGC
NMO1-qF	AGGAGTCAGGCGACAATTCC
NMO1-qR	CTCTTCTTTGCCTCGTCCCT
LHS1-qF	AACCAGCTCGAGGGCTTTAC
LHS1-qR	TTTCTCGAGCTTTGTCCGCT
KAR2-qF	TGGGCAAGAAGGTTACCCAC
KAR2-qR	AGAACGTTGAGACCGGCAAT
MoGAPDH-qF	TCTTCACCACCACCGACAAG
MoGAPDH-qR	CGACTTCTCGTTGACACCCA
OsPR2-qF	TGCTATGTTCGACGAGAACG
OsPR2-qR	GTTGAACAGCCCAAAGTGCT
OsPR5-qF	ATGGAGTTCGTCACTGGGGC
OsPR5-qR	AACTGGGTATCTGCCATGCC
OsCERK1-qF	GCCTTCAATGAGATCCGTG
OsCERK1-qR	GGCACCAAAGTCACTATCTCC
OsCEBiP-qF	CGATCGCTGCCAAGTACG
OsCEBiP-qR	TGAATCCATAGGTGCCATCC
OsPAL1-qF	CCTCCAAGTCGAGCTCCT
OsPAL1-qR	TGATGGTGCCACGGAG
OsPAL4-qF	CTTCACAACAGCTAATCGAG
OsPAL4-qF	CGCACTCCATTTCAGTACCA
OsACTIN-qF	ATCACTGCCTTGGCTCCTA
OsACTIN-qR	CATCTGCTGGAATGTGCTG
